# Differential Expression of mRNAs in Peripheral Blood Related to Prodrome and Progression of Alzheimer's Disease

**DOI:** 10.1155/2020/4505720

**Published:** 2020-10-31

**Authors:** Weishuang Xue, Jinwei Li, Kailei Fu, Weiyu Teng

**Affiliations:** Department of Neurology, The First Affiliated Hospital of China Medical University, Shenyang, Liaoning 110001, China

## Abstract

Alzheimer's disease (AD) is a chronic progressive neurodegenerative disease that affects the quality of life of elderly individuals, while the pathogenesis of AD is still unclear. Based on the bioinformatics analysis of differentially expressed genes (DEGs) in peripheral blood samples, we investigated genes related to mild cognitive impairment (MCI), AD, and late-stage AD that might be used for predicting the conversions. *Methods*. We obtained the DEGs in MCI, AD, and advanced AD patients from the Gene Expression Omnibus (GEO) database. A Venn diagram was used to identify the intersecting genes. Gene Ontology (GO) and Kyoto Gene and Genomic Encyclopedia (KEGG) were used to analyze the functions and pathways of the intersecting genes. Protein-protein interaction (PPI) networks were constructed to visualize the network of the proteins coded by the related genes. Hub genes were selected based on the PPI network. *Results*. Bioinformatics analysis indicated that there were 61 DEGs in both the MCI and AD groups and 27 the same DEGs among the three groups. Using GO and KEGG analyses, we found that these genes were related to the function of mitochondria and ribosome. Hub genes were determined by bioinformatics software based on the PPI network. *Conclusions*. Mitochondrial and ribosomal dysfunction in peripheral blood may be early signs in AD patients and related to the disease progression. The identified hub genes may provide the possibility for predicting AD progression or be the possible targets for treatments.

## 1. Introduction

Alzheimer's disease (AD) is a chronic neurodegenerative disease with an insidious onset and progressive development. It is characterized by comprehensive dementia manifestations such as memory impairment, aphasia, apraxia, and loss of recognition. AD, affecting a large number of people and families, places a heavy burden on society and the economy [1].

With continued improvements in healthcare and the increase in the human lifespan, the proportion of older people in the population continues to increase [2]. Mild cognitive impairment (MCI) is an intermediate state between normal aging and dementia that occurs in elderly individuals. A considerable portion of MCI patients may convert to dementia, including AD, vascular dementia, and mixed dementia. Some MCI patients tend to be stable or even revert to a “healthy” status [3]. AD is the main conversion destination, with a conversion rate from MCI to probable AD of approximately 10-15% per year [4]. Poor life quality was observed in patients with late-stage AD, and the mortality rate is about 25% within 6 months [5]. Unfortunately, advanced AD patients, with no effective medications, always lose their independence and require continuous care. However, the mechanism of AD progression is still unclear.

Various measurement modalities have been studied to predict the conversion, including structural magnetic resonance imaging (sMRI) [6], PET-CT [7, 8], APOE genotype [9], and cerebrospinal fluid biomarkers [9–11]. As the limitation of the accuracy of a single examination, combined measures are recommended [9, 12]. However, due to the limitations of the analytical technologies in different tertiary centers, examination costs, and the acceptance of invasive examination, combined measures are not widely used. Therefore, biomarkers that could recognize patients with a higher risk of conversion would help in monitoring and starting early treatments. Some blood biomarkers were reported to be involved in the conversion from MCI to AD in previous studies, including brain-derived neurotrophic factor (BDNF) [13, 14], beta-secretase 1 (BACE1) activity [15], homocysteine (HCY) [14], and oxidative stress biomarkers [16]. Besides, some biomarkers are related to the progression of AD. Tumor necrosis factor-related apoptosis-inducing ligand (TRAIL), neprilysin, and insulin-degrading enzyme are related to the severity of AD [17, 18]. In clinical practice, possible biomarkers which might indicate the progression possibility may help guide the future treatment and care settings for AD patients.

In this study, we extracted gene expression data from the Gene Expression Omnibus database and obtained blood sample data from AD, MCI, and advanced AD patients. By comparing the groups of differentially expressed genes (DEGs), we obtained and analyzed the genes related to MCI, AD, and advanced AD.

## 2. Materials and Methods

### 2.1. Microarray Data

Microarray datasets GSE63060 [19] with 329 blood samples and GSE97760 [20] with 19 blood samples were downloaded from the Gene Expression Omnibus (GEO, http://www.ncbi.nlm.nih.gov/geo). The GSE63060 dataset contains 145 AD samples, 80 MCI samples, and 104 control samples, as shown in Table 1. The specimens were obtained from whole venous blood and collected from patients who had fasted 2 hours before collection [19]. The GSE97760 dataset contains 9 advanced AD samples and 10 age-matched controls. Peripheral blood samples were collected via the median cubital vein [20].

### 2.2. Conversion of Raw Data

GEO2R (http://www.ncbi.nlm.nih.gov/geo/geo2r) was used to convert the raw data into a recognizable format. Based on GEOquery and limma R packages from the Bioconductor project, GEO2R performs comparisons on original submitter-supplied processed data. DEGs were analyzed by GEO2R. The data were screened with *P* values after adjustment (adj. *P*. Val) < 0.05 and ∣log2‐fold change (logFC) | >0.5 as cut-off values. Probes without corresponding gene symbols were removed. Repeated probe sets were deleted with only one left. Venn diagrams were used to identify and visualize the intersecting DEGs.

### 2.3. KEGG and GO Enrichment Analysis

The Database for Annotation, Visualization and Integrated Discovery (DAVID) Bioinformatics Resources 6.8 (https://david.ncifcrf.gov/) is an online tool that provides a comprehensive set of functional annotation. Kyoto Encyclopedia of Genes and Genomes (KEGG, https://www.genome.jp/kegg/) is a database for understanding high-level functions and utilities of the biological system. Gene Ontology (GO) describes the biological domain concerning three aspects: molecular function, cellular component, and biological process. DAVID and Enrichr (https://amp.pharm.mssm.edu/Enrichr/) were used to perform the enrichment analysis. *P* < 0.05 was considered statistically significant. The results were visualized with R and Enrichr.

### 2.4. Protein-Protein Interaction (PPI) Network Construction and Module Analysis

STRING (version 11.0, http://string-db.org) was used to analyze the interaction of proteins coded by the DEGs. Cytoscape software was used to construct the PPI network, and an interaction with a score of combination > 0.4 was considered statistically significant. The plug-in Molecular Complex Detection [21] (MCODE, version 1.6) in Cytoscape was used to find the densely connected regions and modules. The criteria were as follows: scores > 5, degree cut‐off = 2, node score cut‐off = 0.2, max depth = 100, and *k*‐score = 2.

### 2.5. Hub Gene Selection

The plug-in CytoHubba [22] (version 1.6) in Cytoscape was used to explore hub genes in the PPI network. Top 10 nodes ranked with the degree were chosen as hub genes.

## 3. Results

### 3.1. Identification of DEGs

**Figure 1:**
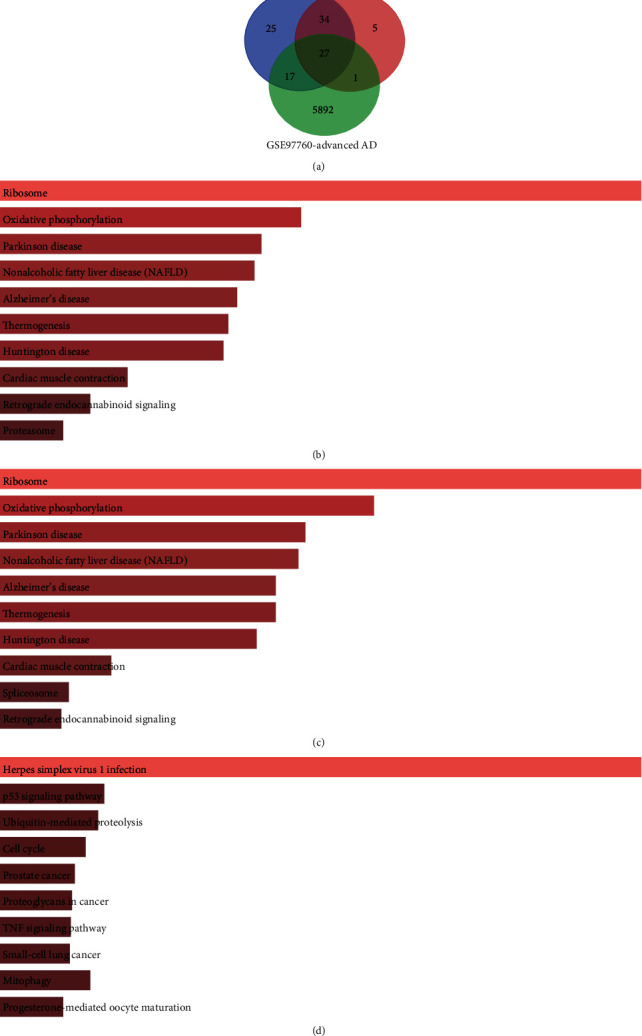
Venn diagram and enrichment of DEGs: (a) Venn diagram of DEGs from AD, MCI, and advanced AD groups; (b) KEGG pathway analysis of DEGs in the MCI group; (c) KEGG pathway analysis of DEGs in the AD group; (d) KEGG pathway analysis of DEGs in the advanced AD group.

After the microarray data were analyzed using the GEO language analysis tool GEO2R, three groups of DEGs were identified. After screening the genes, we obtained 103 DEGs from the MCI group, 67 DEGs from the AD group, and 5937 DEGs from the advanced AD group (Supplementary Table 1). The overlap in the Venn diagram (Figure 1(a)) among the three sets of DEGs contained 27 DEGs (Supplementary Table 1).

### 3.2. KEGG and GO Enrichment Analysis for Intersecting DEGs

**Figure 2:**
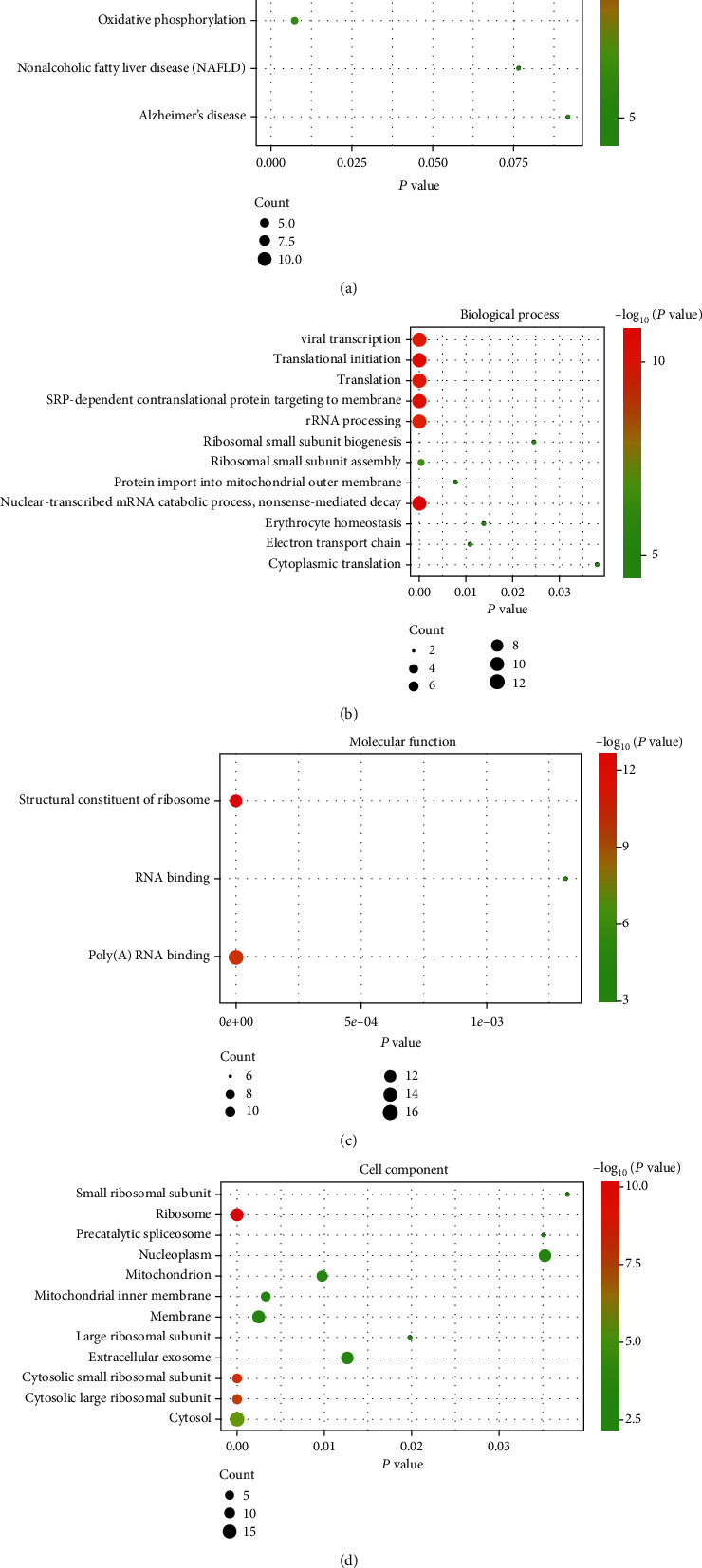
KEGG pathways and GO enrichments: (a) KEGG pathway analysis of the intersecting genes of the three groups and (b–d) GO enrichment analysis of the intersection genes.

DAVID and Enrichr were used to perform the functional and pathway enrichment analysis to investigate the biological classification of the DEGs. KEGG pathway analysis revealed that the DEGs were mainly enriched in ribosome, oxidative phosphorylation, Parkinson disease, nonalcoholic fatty liver disease (NAFLD), and Alzheimer's disease in the MCI group and AD group (Figures 1(b) and 1(c), Supplementary Tables 2 and 3) and in herpes simplex virus 1 infection, p53 signaling pathway, ubiquitin-mediated proteolysis, cell cycle, and prostate cancer in the advanced AD group (Figure 1(d)). Most of the pathways calculated from the DEGs in MCI and AD groups were the same. KEGG pathway analysis of the intersecting DEGs (Figure 2(a)) showed that the main enrichments were in ribosome and oxidative phosphorylation. Related genes in the KEGG pathway are shown in Table 2. GO enrichment analyses were shown in three different domains. In biological process analysis (Figure 2(b)), the intersecting DEGs were enriched mainly in translation, nuclear-transcribed mRNA catabolic process, nonsense-mediated decay, translational initiation, SRP-dependent cotranslational protein targeting to membrane, translation, and viral transcription. In molecular function analysis (Figure 2(c)), the intersecting DEGs were enriched mainly in structural constituent of ribosome, poly(A) RNA binding, and RNA binding. In cell component analysis (Figure 2(d)), the intersecting DEGs were enriched in ribosome, cytosolic large and small ribosomal subunit, cytosol, and mitochondrial inner membrane. The enrichment analysis of the intersecting genes between MCI and AD is shown in Supplementary Figures 1A–1D.

### 3.3. PPI Network and Module Construction

**Figure 3:**
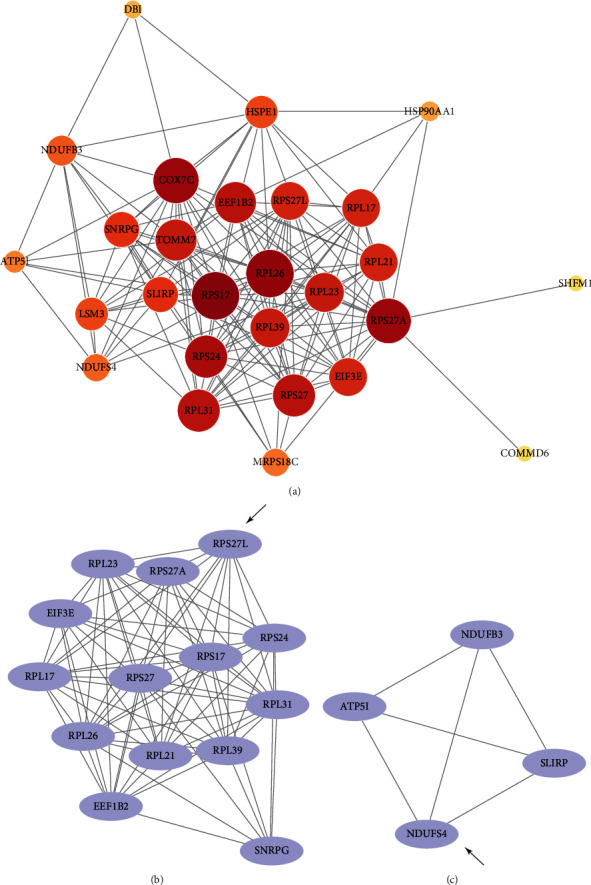
PPI network and modules of intersecting DEGs. (a) The PPI network was set up by STRING. (b, c) Modules calculated by MCODE. The seed genes were indicated by arrows.

STRING was used to calculate the intersecting DEGs to set up the PPI network (Figure 3(a)). The significant modules were calculated by the plug-in MCODE in Cytoscape (Figures 3(b) and 3(c)). As shown in Figure 3(b), the module had 14 nodes and 84 edges. This was the most significant module. The module in Figure 3(c) had 4 nodes and 6 edges.

### 3.4. Hub Gene Analysis

Top 10 hub genes were selected by CytoHubba in Cytoscape ranked with degrees. The gene list with gene names, abbreviations, functions, and scores calculated by CytoHubba is shown in Table 3.

## 4. Discussion

In the present study, we extracted the data from GSE63060 and GSE97760 and obtained three different groups of DEGs. After comparing these three groups of DEGs, we obtained the DEGs related to MCI, AD, and advanced AD. After screening, 27 genes were obtained in the intersection among the three groups of DEGs. Using GO and KEGG analyses, we found that these DEGs were related to the functions of mitochondria and ribosome. By screening the hub genes and seed genes, we constructed a profile of genes that may relate to prodrome and progression of AD.

Predicting the conversion from MCI to AD has always been a hotspot for research [29, 30]. Combined multiple examinations would increase the accuracy of early diagnosis [9, 31–33]. However, the cost and acceptance of invasive examinations may limit the application. Numerous studies have indicated that disease-related changes could be detected in blood samples [34–36]. Some possible biomarkers were investigated in previous studies. Decreased levels of serum BDNF and APOE *ε*4 carriers and reduced hippocampal volume indicate the progression of MCI [13]. The activity of plasma BACE1 is increased in MCI patients who convert to probable AD [15]. HCY and BDNF levels in MCI patients with the APOE *ε*4 genotype may help in predicting the conversion from MCI to AD [14]. In the present study, the changes in MCI and AD were found in peripheral blood. If high-risk patients could be screened with blood biomarkers first, then more effective examinations could be better targeted. AD progression induces decreased quality of life of AD patients. Early detection of the disease progression may guide the treatments for the caregivers and preparations for families. Some potential serum markers were reported in the previous studies. The serum level of tumor necrosis factor-related apoptosis-inducing ligand (TRAIL) is decreased in late-stage AD [17]. Neprilysin and insulin-degrading enzyme levels are increased and related to the disease severity in AD [18]. The serum clusterin level is a possible biomarker in an APP/PS1 transgenic mouse model in the late stage of AD [37].

By grading the degrees of the intersecting DEGs, hub genes were identified. COX7C and TOMM7 are mainly related to the function of mitochondria. A cluster including RPS17, RPL26, RPS27A, RPS24, RPL31, RPS27, and RPL23 is associated with the function of the ribosome. Among these genes, we found that COX7C, RPL26, RPS17, RPL31, and RPL23 were related to AD in previous studies [23–25]. Impaired energy metabolism was found in AD, and mitochondrial dysfunction was the main cause [38, 39]. COX7C encodes subunit VIIc of cytochrome c oxidase which is the terminal component of the mitochondrial respiratory chain. Decreased expression of COX7C has been detected in the entorhinal cortex in AD stages V-VI, while mitochondria with reduced activity were detected early in stages I-II [25]. In our results, COX7C was collected as hub genes which may suggest that mitochondrial dysfunction in patients with cognitive impairment is a sign of disease aggravation. RPL26 encodes a ribosomal protein that is a component in the 60s subunit. In specimens of entorhinal cortex layer II of AD, an apparent decrease in the ribosomal protein RPL26 is observed [24]. RPS17 and RPL31 are upregulated in AD with a rapid clinical course (rpAD) [23]. Decreased synthesis of RPL23 was observed in a tau transgenic mouse model [28]. These different trends may suggest that the expression of these genes may be tissue or region specific.

Several pathways were calculated from the intersection of DEGs by bioinformatics software in the present study. The first pathway was the ribosome pathway. Ribosomes perform protein synthesis, and malfunction of ribosomes will affect the translation of mRNAs. Ribosome dysfunction is an early sign in AD, and both MCI and AD autopsies present impairment in protein synthesis [40]. Previous studies found that pathological tau specifically decreases ribosomal function and causes memory alterations [41, 42]. Ribosome-related protein dysregulation may cause alterations in translation [43], which may occur as an astrocyte-specific event in AD pathogenesis [40, 44].

The second enrichment pathway is the oxidative phosphorylation pathway. Mitochondrial dysfunction, including decreased glucose metabolism, mitochondrial enzymatic failure, and increased ROS production, affects AD progression. As mitochondria are the main provider of energy to the brain, dysfunction of mitochondria will cause damage to neurons [45]. Dysfunction of dynamics of mitochondria may present in the early stage of AD [38, 46], suggesting that mitochondria may become a potential target for the early treatment for AD [47]. A*β* deposition and tau pathology also affect mitochondrial function [48]. However, some studies found that oxidative phosphorylation malfunction occurs in the patient blood before the clinical diagnosis of AD, which may mirror the changes in the brain [49, 50].

As the results were only calculated from two GEO datasets, the study has limitations. The results would be affected by many factors, such as numbers of samples, different clinical data, and different research centers. Therefore, more clinical verification and further research on the underlying mechanism are needed.

In conclusion, the present study found several key genes that showed the potential role of a blood gene expression profile related to MCI, AD, and advanced AD. These data provide the clues as to ribosome and oxidative phosphorylation pathways that might be compromised in MCI and AD and in progression to worse cognitive status. This may provide a noninvasive method for the early recognition of AD and AD progression or provide possible targets for dementia therapy in the future.

## Figures and Tables

**Table 1:** GEO microarray datasets.

	Control	MCI	AD	Advanced AD	Source	Platform
GSE63060	104	80	145	/	Illumina HumanHT-12 V3.0 expression beadchip	GPL6947
GSE97760	10	/	/	9	Agilent-039494 SurePrint G3 Human GE v2 8x60K Microarray 039381	GPL16699

**Table 2:** Related genes in KEGG pathways.

Pathway	Genes
Ribosome	RPL17, RPS27, MRPS18C, RPL23, RPS17, RPL31, RPL21, RPL26, RPS27L, RPL39, RPS27A, RPS24
Oxidative phosphorylation	NDUFB3, NDUFS4, COX7C, ATP5I

**Table 3:** Top 10 hub genes ranked with degrees.

No.	Name	Full name	Score	Function
1	RPS17	Ribosomal protein S17	20	Encodes a ribosomal protein which is a component in the 40s subunit; upregulated in AD with a rapid clinical course (rpAD) [23]
2	RPL26	Ribosomal protein L26	19	Encodes a ribosomal protein which is a component in the 60s subunit; downregulated in entorhinal cortex layer II of AD [24]
3	RPS27A	Ribosomal protein S27a	18	Encodes a ribosomal protein that is a component in the 40s subunit and belongs to the S27AE family
4	COX7C	Cytochrome c oxidase subunit 7C	18	Encodes subunit VIIc of cytochrome c oxidase; downregulated in the entorhinal cortex in AD stages V-VI [25]
5	RPS24	Ribosomal protein S24	17	Encodes a ribosomal protein which is a component in the 40s subunit; mutations of RPS24 induce Diamond-Blackfan anemia [26]
6	RPL31	Ribosomal protein L31	16	Encodes a ribosomal protein which is a component in the 60s subunit; upregulated in AD with a rapid clinical course [23]
7	EEF1B2	Eukaryotic translation elongation factor 1 beta 2	16	Encodes a translation elongation factor that is involved in the transfer of aminoacylated tRNAs to the ribosome
8	RPS27	Ribosomal protein S27	16	Encodes a ribosomal protein that is a component of the 40s subunit and belongs to the S27e family
9	TOMM7	Translocase of outer mitochondrial membrane 7	15	Encodes the protein as a subunit of the translocase of outer mitochondrial membrane (TOM); an accessory protein of the TOM complex [27]
10	RPL23	Ribosomal protein L23	15	Encodes a ribosomal protein that is a component of the 60s subunit; decreased synthesis of RPL23 was observed in a tau transgenic mouse model [28]

## Data Availability

The microarray data supporting this article are from GEO datasets, which have been cited in our article. The processed data are in Supplementary Table 1.

## Supplementary Materials

Supplementary 1 Supplementary Table 1: gene list of DEGs of AD, MCI, and advanced AD groups. The intersection genes among AD, MCI, and advanced AD groups.

Supplementary 2 Supplementary Table 2: KEGG pathway analysis of DEGs in the MCI group.

Supplementary 3 Supplementary Table 3: KEGG pathway analysis of DEGs in the AD group.

Supplementary 4 Supplementary Table 4: KEGG pathway analysis of DEGs in the advanced AD group.

Supplementary 5 Supplementary Figure 1: the enrichment analysis of the intersecting genes between MCI and AD.
