# Supplementary material for: Differential Expression of mRNAs in Peripheral Blood Related to Prodrome and Progression of Alzheimer's Disease
Source: Biomed Res Int. 2020 Oct 31;2020:4505720. doi: 10.1155/2020/4505720 (PMC7648929; doi:10.1155/2020/4505720)
Supplement: Supplementary 1 — Supplementary Table 1: gene list of DEGs of AD, MCI, and advanced AD groups. The intersection genes among AD, MCI, and advanced AD groups. [file 4505720.f1.docx]

Supplementary table 1

| Groups | Number | Genes |
| --- | --- | --- |
| AD | 66 | COX17 ZMAT2 RPL11 EIF1AY MRPL51 ATP5L RPS25 RPS17 AIF1 TXN RPL23 PSMA4 NDUFA1 ENY2 HSPE1 DBI ATP5O LSM3 RPL39 SNRPG RPL17 RPL26 NDUFS5 RPL21 SHFM1 UQCRHL CLC DNAJA1 ANXA1 ATP5EP2 RPL36AL NDUFS4 UQCRQ NDUFA4 SARNP SF3B6 COX7C CD3D ATP5I COMMD6 EEF1B2 UQCRH TMA7 TBCA RPS27L ATP5J SLIRP CMTM2 RPS27A KLRB1 NSA2 PFDN5 GIMAP7 HSP90AA1 GMFG TOMM7 NDUFB3 S100A8 COX7A2 MRPS21 MRPS18C EIF3E RPL31 HINT1 RPS24 RPS27 SNRPD2 |
| MCI | 103 | RPS3A VPS29 MRPL3 DPY30 LAMTOR3 PSMA6 IFIT1 TNFAIP6 RWDD1 TAX1BP1 LY96 CASP1 ATP5F1 RPL35 TMCO1 S100A12 ZNHIT3 DPM1 TPT1 PGGHG COX16 ATP6V1G1 AP1S2 RPL9 PDCD10 S100P RSL24D1 GZMA CD52 UBE2E1 COX7B RPL5 NDUFB2 RPL41 PSMC6 RPS15A EVI2A ATP5C1 SRP14 FCER1A RBX1 TXNDC17 RPS25 RPS17 AIF1 TXN RPL23 PSMA4 NDUFA1 ENY2 HSPE1 DBI ATP5O LSM3 RPL39 SNRPG RPL17 RPL26 NDUFS5 RPL21 SHFM1 UQCRHL CLC DNAJA1 ANXA1 ATP5EP2 RPL36AL NDUFS4 UQCRQ NDUFA4 SARNP SF3B6 COX7C CD3D ATP5I COMMD6 EEF1B2 UQCRH TMA7 TBCA RPS27L ATP5J SLIRP CMTM2 RPS27A KLRB1 NSA2 PFDN5 GIMAP7 HSP90AA1 GMFG TOMM7 NDUFB3 S100A8 COX7A2 MRPS21 MRPS18C EIF3E RPL31 HINT1 RPS24 RPS27 SNRPD20 |
| Advanced AD | 5937 | AP1S2 LAMTOR3 COX16 RPS3A RPL9 LY96 PSMC6 MRPL3 RPS15A RPL35 EVI2A TPT1 COX7B S100P RSL24D1 RWDD1 UBE2E1 RPS17 NDUFS4 RPL39 MRPS18C NDUFB3 DBI EIF3E RPL31 COMMD6 SLIRP EEF1B2 RPL26 SHFM1 TOMM7 LSM3 SNRPG RPL17 RPS24 HSPE1 RPS27L COX7C ATP5I RPS27A HSP90AA1 RPL21 RPL23 RPS27 COX17  DPM2 FSTL1 C5orf42 ELMO2 CREB3L1 SLMAP SAMD4A HAUS2 FARP1 PAX7 KCNMA1 ZHX3 NARG2 GNL3 APBB2 GCSH KRTAP10-8 GLT1D1 MTERF CYP11A1 ABCC6 CLK4 ZNF799 FRG2 NCF4 IRAK1 ZNF646 RPS18 MTHFD2L ACOT12 SLC10A7 BRIX1 NHLRC3 LMAN1 RAB1B LCORL GALNT3 SUMO1 GP1BA NTRK1 OR7A10 DDB1 DLEU7 SCAF8 NAP1L2 CNGB1 FAM134B CCNI WISP2 RDH10 CPXM2 C12orf66 KLHL14 NOS2 CENPQ CHORDC1 ZAR1L SLC2A5 C2orf49 ZNF446 MLF1IP KBTBD8 ZEB1 ZG16 CDK14 ANAPC4 SOD2 CYTH1 PNPT1 MIER1 CHD9 KCNK3 ZFP161 COX15 XK ARID3C SEMA4D MREG MMS22L THRAP3 KIAA1486 TOB2 PDLIM2 ING3 AK7 BANK1 GSTCD MAX OSBPL8 KNDC1 IGSF3 PPCS MRPL1 TEAD1 PTCD3 FIBCD1 NAT10 CHD1 CTAGE5 TMEM184A SLC22A2 RPS6KA5 ATXN10 NMNAT1 EPT1 LHB CCDC34 PTPN22 CYP2F1 GEN1 KIAA1033 WDR89 SLK EIF4G3 ZNF623 NOL8 USP6NL ABCD4 ROCK2 SNX16 CCDC66 VKORC1 PPP1R15B SLC25A46 LARP7 TRPM6 FEV EED SLC8A1 ATL2 C1QTNF3 KIAA0528 MANEA GRK1 ACBD3 ZNF83 SPAG11B PRDM2 SEL1L3 GDPD5 SCGB1C1 CEP350 KRTAP10-4 WHSC1 RAPGEF6 RNF223 WDR45 NEIL1 ANO5 RBM10 OR8J1 DNAH17 PPIL1 KRTAP5-2 DNAJC15 STAU2 SLFN12 FAM98A RAD23B STK17A ABR CNGA3 ZDHHC6 MFSD8 ZNF257 DUSP4 SSU72 TIAL1 GPR78 WHSC1L1 ROR2 HIST1H1E GSTA2 UFM1 C5orf65 ETNK2 APOE CCDC41 TNNT3 BPIFB2 GYPC FBXO11 NR2E3 FRS2 ST8SIA4 RFXAP GMPR C6orf15 ALG2 TPM3 EPB42 AGPHD1 HSPD1 HIST1H2AD FPGT CETN3 CDK1 N4BP2L2 CCAR1 CCDC43 B3GNT7 C17orf99 SLC6A7 ADH5 SHBG P2RX3 PANX3 GCC2 GAS2L1 WBP4 TOR3A HS2ST1 RFX3 CBLL1 IL1F10 NFE4 TONSL EIF1AX PPM1A SRSF11 INTS1 BCL2 HBZ ARL13B TMEM156 SAMSN1 KIAA1324 C4orf29 ZNF284 SEC14L1 CHFR THADA TNXB TBC1D17 ATP11C CARD8 TRIM49L2 ERBB2IP IMMP1L RSRC1 NIPSNAP3B CXorf56 FBXO24 THRB RNF39 MMP15 MTMR14 ELAC2 DNAL4 TMEM59 SEC23IP CNOT1 SNX6 ZBTB11 CEP120 ZNHIT2 SPTBN1 RICTOR RAG1 HSF2 CDC42EP3 SRP72 TSPAN7 PTPN5 OR4D10 MFN2 SNAPC1 ATR GNA11 TLCD2 TACR1 THAP1 FAM204A UPF3A SNX25 CDK17 DPYD TTLL4 MYO5C ANKRD49 POF1B METTL21A KDM2A CACNB1 LDHAL6A SETMAR QKI NUDT12 KDM6A OFD1 C14orf149 PDP1 SLC22A14 GPHB5 DAPP1 ATF7 LRRC10 C17orf85 FGF1 IL6R UFL1 COL7A1 CDC14C ASNSD1 SPG20 SEZ6 BAGE4 NANOS3 GCA ECEL1 CLDN6 FANCC L2HGDH ZNF655 GPATCH2 MCOLN2 NQO2 ARAP1 PLCXD2 AGPS ZBTB20 MED14 HP1BP3 ENHO SPRY2 PRSS56 LZTFL1 C6orf105 KLHL7 SUV420H1 DCUN1D5 HDHD2 GMFB CCR3 IGDCC3 CDHR5 ANKLE2 SPATA4 AGTPBP1 PRR5 NOP58 DOCK11 DNHD1 PTPN2 LONRF2 C19orf67 C14orf166B GIPR AHI1 SLC3A1 GORAB PIGF UBE2W AHNAK KIF14 ACTR5 KRT15 C10orf99 FAM175A YIPF2 HEATR5B FHL1 SMC6 RTN4RL1 GATA1 IL17C ETV2 DUSP18 PSMD5 OR2W5 RSU1 AHR MAD2L1 GRASPOS ATP6V1C1 CHMP2B RPS21 CAMSAP1 XCR1 LUZP6 COQ10B UBE2N SORBS2 PSMF1 C7orf60 SH3PXD2A SPINK5 CYP4A22 KLKB1 C16orf71 RBBP6 IFI27 KRCC1 SYTL5 TJP3 MAGOHB MOB1B PARG OLFML2A OR2B2 C17orf77 ZNF667 TFEC SFRP2 RG9MTD1 RIC3 DCX ART1 HIST1H4I GGN ZNF709 ZNF738 OTOA EXOC1 PDAP1 BRD4 ZNF146 ZAN ARFRP1 CRYBA1 NPFFR2 MTMR6 ARGFX UBTD1 SOX9 CASC4 PCDHGB4 CUL4B TNRC18 AKIRIN1 OR56B4 VRK2 RDX PACSIN3 ZNF568 JUND ITSN1 SVIL C19orf2 DCTN1 CELF5 TNKS2 CXorf23 TTC3 TCF21 JPH2 AMELX PAQR3 NECAB2 ANKRD26 TCP1 SEPT12 CMA1 CENPL PTCRA VWCE ZNF419 PAWR TLK1 TBC1D3B TIGD2 SCRN1 TAB2 FAM160B1 DDI2 HPS1 UCN2 ND6 FAM161A TFE3 ZMYM5 ZNF586 POLR3G DCT ZNF726 KLHL28 TANK PGLYRP1 C1orf49 RCAN1 IL8 SET DNAJB4 HIST1H4L FAM120A SLC38A9 SERP1 SHH TACR2 C6orf225 EIF2S1 TNFRSF17 RBM7 JAK2 DPYSL4 TSHZ1 TAF9 BICD1 RAB5A METTL5 DCAF11 PRRT1 PLEKHM3 ENKUR ANKDD1A JRK XPO4 ICA1L FAM131C XIAP HCAR2 TLX1 OR2M2 BDP1 LOC391766 RPS29 GPR174 CCDC60 MKLN1 TALDO1 OSM NF1 IMPAD1 SQLE OPN3 TAOK2 PPFIBP1 GLIPR2 EIF5A NEGR1 YTHDC2 NCF1 ZNF614 MAP4K3 NUP133 INPP5B GRPEL2 FIS1 UBE2J1 IL20RA HNRNPK CACNG2 FAM157A ELF1 FAM71A CLPS GCFC1 BTBD9 FOXN2 PARP4 C19orf22 GK5 IGFBP2 DNAJC25 FAM72D KLRG1 ZSCAN12 ANP32A MGP CLDN10 TRPC1 CCDC144A MEX3D SMS ERCC8 TAF1D GPX4 SYNGR4 OR51G2 STRN3 FBXO3 CMKLR1 TMOD2 PEF1 FAM199X C9orf30 VPRBP ZNF750 SLC44A5 PPDPF MELK ZNF681 RABGGTB IL15RA PTP4A3 KCNK7 HMBOX1 CUL3 PAGE2 CCT6B SNCA GPX2 ITK FAM122B CLIC5 C2orf53 ZNF551 ESRRB SLC48A1 NR1D2 HBQ1 DNAJC4 KLRC4 GEMIN6 C13orf27 BSDC1 ARSK CPNE9 MEF2B MAP2K4 PTPN4 NADKD1 FOLR2 STX5 LYZL6 CD72 ZNF480 ERH SRRD PROS1 SOCS4 HN1 SLC16A11 MAS1L PPP6R2 APOL1 TBCCD1 SETBP1 CDH11 NDC80 ZBTB41 ZBTB7C AHCTF1 AP2S1 MME C2orf69 GOLGA6L9 KANK4 CCDC14 ZNF713 THEG KCTD2 SNRNP48 NRXN1 SPTLC3 DTWD2 CPO AGBL1 CCNA2 LCE1E CGRRF1 MCAT ARID1B ZFP1 C12orf48 CEMP1 E2F7 WDPCP NIPA2 SLC23A3 SLC35A1 LAMA3 GPR160 GAL SLC14A2 RAVER1 C6orf170 SRSF3 SP4 PCYT1B SLC25A39 C14orf129 C10orf114 KIF1B MBD2 RPL36A-HNRNPH2 AMOTL2 C6orf120 SPHAR FOXO1 KIF5C C17orf62 PANK3 GPM6A CRLF3 OR5W2 CLCNKB WDR82 MAPK1IP1L USP51 AMPD3 NPHP3 GYLTL1B FBXO7 CEP104 TBC1D3 LRRFIP1 ZC3H14 CNOT2 MAN1A2 IKBKAP FKTN TWSG1 LOC643802 DDX50 ADAMTS6 STYXL1 RPP30 TRIM16 CDC40 STMN1 BLVRB SLAMF6 SETD3 DPP6 PQLC3 MKKS ELK4 STK39 MMP24 ZNF846 SFTPA1 CKS2 RHBG SMURF2 CR1 HMBS ADAD1 SLC25A24 C20orf112 EFR3B GPBP1 CCDC129 ACER3 PES1 KLHDC1 FAM84B ZNF287 DAAM2 AVL9 RFK ZFYVE9 TRIM24 OR2AG1 SLC25A30 FBRS SLC22A5 KRTAP5-11 GRIN3A OR2T4 DEPDC1 CHTOP C10orf47 SEPT7 PRSS42 PTCD2 GNLY GRAMD1C ZNF165 USP38 ANKRD65 ZDHHC17 KIAA1429 ARMCX3 VNN3 YBX1 PHAX AK1 STK24 SPEG CLNK TBC1D24 ZNF695 SSX3 KIAA1045 ABCD3 C1orf183 MAGEA8 C4B MTF2 DGCR8 BOD1L PARVG ZNF676 HIST1H4B FIG4 HES2 SP7 FAM3C EIF2AK2 AP1S3 UCKL1 LMO7 DCST1 HNRNPH1 OBSL1 ZNF84 KRTAP10-5 PPP1R15A C12orf11 C1orf96 C12orf49 MDC1 METTL18 ANKRD18B NKAIN1 HTR1A SIAH2 ATP11B IFNE ALOX12 CCDC103 RB1CC1 FSD2 RPE EIF4E EXOC4 LALBA RMND5A TCHP HEATR5A EXOC3L4 ZNF385C IMPG2 SLC29A4 BATF2 SLC35F5 EMB ZFAND1 KRTAP1-3 KIAA0408 SHE F13A1 IFIT1B BRCA2 ACADM KCNQ4 XPO6 LCE1A FMR1 CCDC117 CHMP2A BBS9 USP15 CR1L C9orf72 FNDC9 SDSL ERCC4 ESAM PLA2G12B HAUS1 FAM83B MT1E IFT172 ST7L GFOD1 CNTD1 C9orf153 GHITM CCDC3 TMEM182 C9orf21 SH3TC2 IL10 AGRN WDR33 CEP290 RTN2 HIST2H3A GOLGB1 LCLAT1 TSKU ETV3 OR8H1 CHD7 C10orf62 SLC30A8 RNF126 PSMB11 GPSM1 CLIC4 HIC1 ACR NKX2-3 GP1BB ANKH API5 TNK1 THUMPD3 MOV10L1 BIRC6 SLC8A2 ITGAD SH3PXD2B SAMHD1 PTBP3 EPC2 ARL17B C20orf85 SPRY1 FAM117A MYSM1 TRIM4 SH3YL1 SERPINI1 SNX18 MDM2 TDRD3 ITGBL1 EID2B IPPK BIVM KLHL15 NBEA CXCL2 ZMAT1 CPXM1 SESN1 APPL2 TNPO1 MRPL13 CLASP1 METTL6 RAB6A NCAM2 PAPOLG CHML CDK13 OC90 SIRT5 ZFP36L1 CYSLTR1 TMX3 FXYD5 TWISTNB TIAM2 OR4C3 NKX3-1 CHD2 HIST1H2AJ ZNF10 PURA PCNP CELA3B GTF2IRD2B CREB1 HOXC4 FANCM NAA38 DDX3Y LGALS3 ASPDH CFL2 RASA2 ZNF878 LRRTM2 C4orf32 STAG1 NAP1L5 KCNA3 BTBD1 SKIV2L2 SERPINA11 FCF1 APOOL CD28 DIABLO ZNF624 SEPT2 LRP8 TGFBRAP1 MASP2 ZNRF3 GPATCH3 AGL ZDHHC20 NFE2L3 SRRM5 TMEM181 MINPP1 MPHOSPH6 TNP2 STK31 EML4 LOC100289187 ATP6V0C TTC39B KCNG3 SSB TMC5 SCT FKBP3 SKI GLIS3 LRRC70 TMEM50B TCERG1 MPP6 TBL1XR1 SZT2 ANKRD36 RPRD1A TNFSF8 TMEM126A GABRP DNAJC5B CELA2B PDE5A EIF3J HBE1 CPA3 PVALB TMEM167A MFN1 CPEB4 F10 TCTEX1D4 CAMSAP2 TAL1 HCFC2 ABL1 PTPRG PLEKHG5 MOCOS OR7E24 TXNRD1 SLC30A1 SRGAP1 LY6G6F PLAC1 LRRC26 C8orf31 SLC6A8 MKL2 CHRFAM7A MBTD1 CWC22 DCAF4L2 FUT11 USP33 PPP1CB DIO2 C15orf39 LRRCC1 CCT3 ZNF121 SLC5A10 OR13A1 RAD50 PIWIL2 TRIOBP SYNE1 MBTPS2 PSMD10 APLN NCOA1 WDR44 HRH1 C5orf30 RASGRP3 CHEK2 C19orf46 KBTBD10 IL28A CCDC75 CD247 MED21 KIAA1239 LOC643699 FBXL17 OR2A25 DCAF13 SPTBN2 IFT88 SLC26A2 RNF111 P2RX2 ATP2B4 TARDBP P2RY10 SLC24A1 ARHGAP5 ZNF160 ZNF688 CETP HDAC11 SLC9A4 DNAH10 HBM GAS2 METAP2 RHOQ TNIP1 STXBP4 PAN3 TCTE3 CCDC85C PDHX ZBED4 KNG1 GNG11 ZNF720 CLCN3 XRN1 GPAM CANT1 CDK2 ZER1 FCRL5 ZNF99 INTS2 LRP12 SNX22 SDC3 OGFOD1 MSMO1 ZFYVE16 CCZ1 HIST1H2AB SYNJ2BP MECOM POLE E2F2 DDX17 PDE4DIP COL4A6 LRRC1 NAB1 CATSPERB PRAC C4orf49 ZNF559 NADK TRPM3 MED31 MCF2 ZMYM4 MAFIP ZNF532 RAP2A FECH TYR AZIN1 TMEM64 SYDE1 ZDHHC21 UBE2CBP ARG2 LEKR1 BRMS1L NHS CNTNAP5 TM9SF3 TAF8 RNF139 DDHD1 ZNF594 RBM41 GOLGA6L1 SFTPC RGS12 RNH1 C10orf131 TOMM5 CCDC157 TMEM41A PRRG3 OSBP2 LRCH1 MAMDC4 CBX3 C3orf58 CHIC1 ACAP2 PGPEP1L KBTBD2 PPP4R2 UBE2E2 SLC39A10 KIF3A MYL9 ACP1 C1orf194 CDC23 SIGLEC5 ZMYM2 TAB3 RTBDN LRRN4CL NMUR2 B3GNT6 KIAA1586 NCK1 ZNF550 LPL FBXL3 CA5B SPIN3 MPHOSPH9 MAGEE2 CLEC2D SH2D4B FAM157B HIST1H3D CD84 RNF10 RASA1 ETV3L CHADL ZNF567 IMPA1 SH3BP1 ISLR2 FAT1 ZNF660 ERMN CENPB KRTAP4-7 LOC100287509 LOC100652777 C12orf36 HIST1H4H TMEM133 LRG1 KIRREL STAM2 TNAP PIK3R1 GPC3 C4orf34 SV2A MAGEA12 HOXC11 GSG1 C18orf19 HELZ ADCY4 SLC46A3 PTPRJ TET1 ADRB3 MPEG1 DMD LOC100653193 STAMBPL1 ADCY2 HOOK1 ZNF737 TTC14 KLHL5 FAM178B FOXH1 NFYB KCNQ3 HIGD2B ANKRD33B FZD3 SOCS5 C9orf123 WFDC12 TFEB C19orf69 KLC2 ELMOD3 C10orf105 SMARCAD1 ATG12 BMI1 SH3RF1 RNMT MTCP1 SHOX BLZF1 ZHX1 PCDHGB3 RPAP2 ATRNL1 CAV2 CENPK MIA3 TMEM135 KRT33A KDM2B WARS2 NEURL1B DDO SLC25A45 S100A7 ZNF789 ASF1A TOMM40 GNB1 UPF3B TCF23 NOBOX CD200R1 C22orf15 UST LOC100652953 TIGD3 GSTM5 CALD1 NIPAL1 AIG1 ST6GAL1 ARHGEF25 APOA4 PMS1 SKIL RAB28 HMGCS1 ATP2B3 POLR3F RAB10 ZNF570 TLE4 TSTD2 ALAS2 TLK2 OR2M3 ST3GAL5 MARS2 IL28RA CNOT3 IGFBP3 DEK HSF5 TTC9B C12orf23 PSPC1 UPF2 SORBS3 JPH1 ZNF823 KIAA0564 DOPEY1 CPT1A TERF1 ALMS1 LMO1 ACTBL2 EFR3A SUCLG2 SEC61A2 UCP3 UBAP2 TREM1 CDKN2AIPNL OST4 SLC13A3 IFNAR2 BBIP1 NXF5 N4BP2L1 PITPNC1 KCTD3 LONRF1 ANKRD18A C2orf88 CCP110 ODF3B CACNG6 C6orf94 CEP128 CCIN ANKRD7 PYCR2 CRLF2 DOCK7 LIN28A HIST1H1C KRTAP9-9 ABCA5 DNAJB14 TPR MTX2 LYRM7 HIST1H4A SERPINA3 ATPBD4 GOT1L1 WDR3 C15orf54 TTC33 KCNJ16 STAB2 WFDC6 MCPH1 C9orf3 KIAA1432 LBH MYO1D HSD17B14 NFU1 TBC1D23 FAM13B GLP2R HNRNPU R3HDML MAP2K3 DYNLT3 ATXN2L FAM3D SESTD1 OMG SLC4A4 ZNF486 NARS TMEM30C ADAMTSL1 CCDC126 ZNF675 CSNK2A1 ZBTB32 ODF4 NTN3 HORMAD1 AAK1 ZNF682 JRKL MAFF C1orf86 TMOD3 EEA1 AKT3 CRB1 C19orf21 IL1R1 UBR4 HRSP12 KTN1 CTTN ABTB1 UTP15 ARL5A ZNF776 RBAK HEPACAM ITGB5 YIPF3 C10orf96 GGT5 FAM75C2 KCNE4 PARD6B ARHGAP11B MBD1 RRAGB RCN2 HIST2H2BE WDR73 THEMIS KIAA1751 NDUFA5 THAP9 ZBTB40 LYPLAL1 COL12A1 TMEM206 ZNF17 TRIM62 SAT1 ABLIM1 BTAF1 ADAMTS7 ZNF280D RCBTB2 RBMS1 ZNF22 RNPEPL1 NSUN4 RFX2 RDM1 PIGM ZADH2 MLLT4 FANCD2 ACY3 ARL6 HMGB1 DUSP8 ACTR2 ZNF519 MEF2C FAM174B LBR LZTS2 AFF3 MYB KLF3 B3GNT5 C17orf103 TBC1D19 PTGER3 CHPT1 ATOX1 MAP4 KIF26B ACTR3 ZNF416 UGCG ZNF225 C1orf106 ZNF485 LPP ORC3 HIVEP1 HECA ATRX UBTFL1 CHST8 ANKRD34B RYK IL26 HSPB6 MACF1 PPIAL4A MDFIC CHRNE WNK2 EPB41L4A SH2D6 PCMTD2 NUFIP2 MNAT1 ZDHHC2 MBNL2 ADD3 TAF4B ATP6V0D1 SLTM KIAA1279 APOBEC3D RTDR1 MALT1 POLR1C ZNF385D TUBGCP3 ZNF85 NBEAL1 MTM1 CSNK1A1L FAM115C OR10G9 RECQL TFDP2 NLRC4 ENPP4 POLI ECHDC1 RNF170 CPSF4L USF2 GNAI3 KCNE1 FHL3 SLC37A3 CD4 RAD51B MTCP1NB NMS ZNF248 PRR24 ZNF230 FAM66D C5orf24 UGT3A1 IFI27L1 MFSD6 SLC9A5 SH2D1A MUSK ZNF347 TPP2 STRADB GTF2E1 PKD1 ATAD2B CHSY1 AGAP11 TTF2 SFPQ CDK15 RGS16 ZSCAN30 DIAPH2 RTL1 PTPN12 ABL2 CRYZL1 MECP2 PSMA1 PNPLA4 DIP2C PDLIM7 CA14 BMPR1A TIMP3 STAG2 FUBP3 ACACA LRTOMT RGS3 ARL11 MAML2 SPAG16 C6orf192 COG6 ARHGEF12 SEPT10 LYPLA1 SCOC LNPEP CTDSPL2 NAPG ZCCHC4 C14orf45 FBXO22 ZNF669 YIPF4 PTGDR ANKRD10 RERG SLC16A7 CHMP5 FAM192A OR9G9 ZRANB3 NEK4 MRPL19 KRT13 RBBP9 RBM33 YTHDF3 C5orf28 ZBTB38 TMEM30B OTOR KATNAL1 DRD5 UBE2I RAPH1 PLTP CTIF LCE1B C14orf28 MYO6 DNAJA4 LRRC38 ATP11A ARL5B ZFP37 ACRC ARMC1 ZNF154 FAS C3orf64 C1orf63 CNKSR3 HSPBAP1 CSTF3 BORA PRKCI PRTFDC1 LEPRE1 MRPL12 ZNF574 CYP21A2 RASAL1 COMMD2 DDAH1 MGAT1 UGT2B15 TIPARP MIER3 LRRC58 EXD2 LOC388630 PRY2 SLC26A3 KIF11 IPO8 C6orf191 SKP2 SYNC PHC3 PPP1R8 NOVA2 C12orf50 GOLPH3 FAM102B UBLCP1 PIBF1 KIAA1143 LRRC37A2 OPN4 ZNF117 DCAF12 NKRF LMF1 TNFSF15 FANCL KBTBD6 HMGN3 C9orf50 PPAP2C FAM133B PAG1 C12orf76 SOS2 CAPN14 DHDDS C1QTNF5 CCDC99 TNNC2 USP16 APOL4 LARS BCL9 C9orf41 FAM40A ZBTB2 RAB14 TRAF3 ZIM2 C9orf171 USP10 B4GALT1 WDR75 CHD6 ZFAND4 RNF145 FGD4 DYNC1LI2 EFCAB7 ZNF81 ZMYND8 PDXK C9orf163 ZNF670 HSPB11 OR52I1 SPICE1 N4BP2 ZNF318 NDNL2 ZNF609 FCRL3 OR2W3 ARHGAP4 FAM135A ZNF382 DEGS2 ITGA1 ERMP1 TMCC2 ARHGAP33 TCF12 HACE1 ZNF721 MYOT SGIP1 PLEKHG2 PRRG4 WDR5B NCEH1 FBRSL1 ARMC8 BAG1 CEACAM18 VPS36 MYNN AEBP2 ZNF625 C1orf190 CORO2B TBXA2R DEFB105B RNF144A CBR4 CST1 SMC1A TP73 DSC2 RBMS2 TMPRSS6 ANTXR2 SLC6A18 PEX1 BCL9L RET MASTL RUNX1 PRKAA1 SLC22A20 FADS2 IL1RL1 ZNF765 SNX10 C3orf33 CCDC104 RCC2 RNF103 DBF4 C1orf64 CROT MAPK9 ZNF543 SLC12A2 PPTC7 ZNF540 OTX1 ISPD CD58 CSTF2T LRRC25 GRHL2 IREB2 CCDC76 PDCD7 OR1G1 ZNF566 ABCB1 CNR1 SSR3 NAA16 FAM69B TRAT1 CC2D1A CST9 MAGT1 FAM188A C7orf36 FTL DYRK2 RNF2 KLF6 NR5A1 PIK3C2A ABCD2 CYP4V2 LOC728763 SCNN1D SCUBE1 HUWE1 ARNTL2 ZNF595 CD180 FAAH IL17A C22orf25 PROSER1 TMPO LYRM5 ASPH CPA2 HDGFRP3 RRAS2 NBPF6 PKD1L2 NOTCH2NL PDE9A ANKRD42 UBR5 PRPF4B GP6 LEF1 SLC22A8 SEC14L3 PEAK1 METTL10 SLC22A1 EYA1 RASA4 MAPK14 CCNL2 GJA4 GTF2F2 C15orf33 PPP1R42 CDADC1 DPM3 TSPYL2 AP2A1 VBP1 MORC3 TH GPR126 ANKS1B PATE2 PPHLN1 RAPGEF1 KRTAP9-6 MAP3K8 STC1 ALG13 RPAP3 KLHDC8B NAA20 SLC13A2 DDX26B GSN EGR2 SMARCA5 PLEKHA3 PLS1 FRMD6 FAM18A EFNA1 YY1AP1 HOGA1 ANK2 SH3GL3 SENP6 ARPP19 RIF1 ZNF516 PRLH VEGFC DBT C21orf63 CGGBP1 INVS ELN RNF19B KLK2 SAMD8 REG1B UQCRB ANO2 NAA50 TMEM209 MRFAP1L1 KCNK17 KIF21A RHAG ABCE1 UNK ARHGEF3 EXOC8 SUPT3H CSNK1G2 MAML3 SLIT3 PEX13 NPM2 RWDD3 MAN1C1 KRTAP13-4 ZNF518A CD226 PDK2 PHC2 SLC38A2 PPP2R5C SYNJ2 WDR36 ST3GAL6 ZNF578 LRRIQ3 IL34 GPR141 C15orf29 PRR5-ARHGAP8 SPIN4 DDX52 SLC36A4 UBA3 NEK1 MYH11 C12orf24 AGK DENND4A TMEM146 LOC649201 CAP1 EIF5A2 SEZ6L2 SEPT9 SHPRH STX7 ATXN3 ADO CDC42EP2 RABGAP1 SIPA1L1 FBXO5 OXR1 DGKB ZDHHC19 TGFBR1 RNF187 MRC2 KRTAP6-3 SFMBT2 C1orf52 PDC LRPPRC ACVR1C CD97 SETD5 GALNT12 PTER NINJ2 CRLF1 ATP1B2 PRR15L COL4A3BP ANKRD54 TSNAX ZNF497 C12orf60 CCBL2 SLC30A4 ATM C5orf34 PCID2 ODF2L LTN1 KLF12 C18orf62 ARHGEF18 HSPA1A ARRB1 LOC100293516 BLOC1S3 RUNDC3B SUMF2 CENPA TMED5 WBSCR17 MCAM PARP2 NDFIP2 IPMK CDCA7 MAP7D2 USP45 ZNF134 RIMKLB ADAMTSL2 ZSWIM6 SFN CALY FAM190B PLSCR5 DENND5B TNS1 PLCD4 MDM1 KCNH6 OR51T1 LENG8 CCNJL USP53 SPANXB2 CDC20 SCGB3A1 MRPL50 E2F5 SLC26A11 RCAN3 STYX CBWD5 C4orf33 LYPLA2 GPR149 SMAD4 MARCH7 FCHSD2 SLC6A20 SLC4A7 SGMS1 ZNF195 FXYD4 ZNRF2 GART CREBZF PER3 TRIAP1 CACNA1F PCDHGA12 ARMC10 C10orf27 ATG9A RPL6 TMEM40 GJB7 CAMK2N1 NFIC ITPR2 GABRR3 UTP23 HIAT1 RFX8 PELI1 MLLT3 UBE2B MT1X KRTAP25-1 CCNT2 MUC4 OMA1 TMEM63B NUP50 ELMOD2 SULT4A1 WASF3 RQCD1 GLYCTK SRP19 SIRPD TRAPPC10 LEMD3 PLEKHF2 KHDRBS2 RNF138 IRG1 HOXD1 DIMT1 SOAT1 MAGEB5 LOC100132396 HIST1H2AI CSH1 MAP4K5 F8 KPNA5 ACHE PLAA C5orf54 MYO7B ZNF184 SEH1L OASL ADAM21 GDPD1 KCTD9 LCE3B ZNF267 ZBTB6 TSPAN5 PABPC4L AMIGO1 FBXL19 PRNP PDIK1L DARC SLC9B2 ZSCAN1 ZRANB2 FBXL8 HIST1H4K FOXA3 NEB GABPA EIF5AL1 ZNF260 TLR3 CTGF HMGB2 USP37 PTAR1 BRD1 CSRNP3 MTMR9 MCCC2 TRIM58 IGJ TAS2R14 NAA35 TMEM154 MTX3 TOPORS KLHL32 ZMYM1 BCL7C HNRNPC PSTPIP2 CLDN19 ZCCHC14 DNAH2 FXR1 PNLIPRP2 OR8G5 TGDS BCAS4 CASP3 ITGB3BP FAM120C FRG2C ZNF429 HDHD1 ENTPD5 LYPD6 DGKK ZNF461 NCKAP1 CLK1 WDR47 KLRF1 PKN2 PRPF40A ACE ZNF333 SMR3A THAP2 CLDN12 OPN1SW TUBE1 DUSP11 DHFRL1 TIGD7 GFRA3 MGAM MFSD2B PTMA GLIS1 GFM2 ZBTB37 FMR1NB GTPBP8 C8orf85 UHRF1BP1 IVNS1ABP SGPP2 DPT DUSP19 C9orf4 NDUFV3 ACTL8 UBE2NL PPP1R2 ACSS1 UBXN8 PACRG ABCG2 PGAM5 GRB10 CCDC154 KRTAP12-1 TBL1X LOC100133130 LOC100506422 RFWD3 RBM12 LRIF1 H2AFJ EDIL3 INSIG2 PCDHB7 GPR65 ZNF155 FUT1 SMC2 C6orf204 C19orf66 MS4A1 SCYL2 TYW3 PEX2 LRRC52 LEAP2 KRTAP5-1 SLU7 B3GNT8 WDR11 BTN1A1 PAPD5 TMEM65 PCDH1 MRPL32 FDX1L PMEL KLHL33 CAPZA1 CDYL2 HRH3 CAPN13 CRYGD NUS1 DNAJA2 MAOB ITGA2B CACNB4 CLDN5 BST2 CHM PRR21 OR2A12 SERINC5 CCNL1 FAM105A USP6 POMP INPP4B ZNF732 TMPRSS9 NAP1L3 ELK1 HGD C10orf57 BMPR2 KLF7 C17orf58 TCP10L MYO3A C8orf80 GDAP2 ADAM33 MAPK6 MGAT2 FAM198B USP31 SLC5A6 RNFT1 KIAA0907 KCNH8 CNIH2 SUMO2 SDCBP NUCB2 CUL5 ICAM4 FRMD1 FOXO4 ORC2 TAF13 LYG2 TAS2R45 SFT2D3 INA ZNF354B MCOLN1 PSG2 HIBCH YBEY PLA2G5 UBL3 SEPSECS FAM90A10 PSORS1C1 PIGA LPA CD5L ZNF238 LY75 COL4A4 POLR2K IL17REL SASS6 TUBA8 RREB1 PABPC1L COL28A1 ENPP5 KRTAP3-1 AXIN2 DENND1B ZBTB1 TMEM8C C16orf13 SRRM3 TAF1 CXorf57 NDST2 LCE3D FAM173B S1PR1 LRRC72 SAMD14 C20orf7 PION PPME1 PIK3CA ATP6V1E2 NBPF10 IRGM SLC9A6 CKLF DCUN1D1 MBNL1 KDM4C DAB2IP SLC6A5 NUP160 ZFC3H1 DCUN1D4 ERBB3 GSX1 ZNF642 RPL3L DHX57 FAM177B PSAT1 KIFC2 TDG FAM116A PLAG1 IFNG ZNF226 FIGNL1 AGPAT2 CCDC65 C14orf1 SLC6A19 ZMYND17 ENTPD4 GTF2IRD2 CCDC82 TP53INP1 ZNF300 WDR35 LATS1 HTR6 KRTAP10-7 KCNQ2 SPOCK2 SECISBP2L PDZD8 NEMF ZNF841 PEX3 MED1 CLU CCDC33 ATG4C GPR34 RBM11 PABPC4 CXCL12 DDX6 OR10W1 FAM109B C15orf53 ABCB10 IDI1 KCTD6 VPS28 AP3M1 DIAPH1 CEP76 ZNF460 CCL16 C19orf12 COG5 COMMD10 COPS8 SACM1L AKAP9 BUTR1 C17orf42 GPSM3 TOP2A CX3CL1 GPR50 ARAP2 GYPB OXGR1 FAR1 KLK5 SPTB KLRC3 CAPRIN2 SNX30 SF3B1 IPO7 PCM1 CAV1 GNPDA2 ANKRD5 TSNARE1 FMO5 HPRT1 HELLS ABHD3 FAM69A PIGN GALNTL1 BAG2 DBF4B THNSL1 C20orf123 SHROOM3 CCL28 JAM2 FIGNL2 MSI2 LRRC40 SMG5 IL6ST PRSS23 ANK1 FAM172A OTUD6A DCP2 PPM1L NHLRC4 POLR1D GIN1 LRRC37A3 ATP4B KRR1 CDKN2AIP C1D PC SETDB2 SGMS2 MON2 CNTNAP2 RABEP1 USH1G TMEM116 IKBKE HIF1A LGALS9C TRIM71 TMED7-TICAM2 PKMYT1 LCE6A KIF18A TXNDC9 OTUD5 TMEM22 SLC41A2 MAP2 LARP1B LYL1 SLC25A19 IBTK OXER1 C21orf91 KCNIP4 CDC73 FCRL4 ZNF280B GOLGA8F SPRY3 WRN NFATC2 DZIP3 ST14 GDI2 SCML1 FGD5 REXO1L1 USP17 TMEM201 SELT ETAA1 NPIP MLLT10 COX6B2 PHF14 GOLGA6C TDGF1 NRIP3 TMX1 WDR76 NKX1-2 BAZ1A TRIM10 EFTUD1 GCNT4 OTOGL CLEC12A MAP9 GPRASP1 ACBD4 MRS2 CDKN1C CXorf36 DSCR3 ZFAND3 C7orf43 HSPH1 MBLAC2 AQP1 TCERG1L NIPA1 IL7R ACTB FAM83F UBA2 LYNX1 PRSS16 KIF13B WFIKKN2 SUGP2 RAB12 KRTAP4-1 C1orf226 ANKRD12 PCDH9 DDX51 ZNF626 MKRN3 ZNF295 HEXIM2 SLC30A5 ACVR2B TTYH1 ZNF517 IYD ARHGEF33 HMGCLL1 C18orf25 UBQLN1 LIN37 ZNF587 GLB1L3 XKR6 HIST4H4 RANBP10 MYO7A GPR142 DOCK5 SLC41A3 DSEL UBA5 TMEM128 FBXO4 MOS NOX1 C11orf36 CSNK1G3 SRFBP1 MBIP C9orf96 TMOD1 RNF219 PRELID1 ANGEL2 RBMX IL33 GLIPR1 UBXN6 CIZ1 ZNF700 WWTR1 MARK4 DDX60L METTL4 RNF212 ZNF680 MDN1 C9orf68 FOXN1 SLC35A3 AMOTL1 CLEC16A IER3IP1 HERC4 SLC38A1 RGP1 SYNJ1 KLK10 PHF20L1 ZNF98 ST3GAL3 RAB11A PTBP2 TCF7L2 C6orf228 ME3 CHD5 ZNF431 CACYBP TYW1B GPD2 SETD7 ZNF800 ITGA9 ZFR GPX5 QSER1 TOR1AIP2 ULK2 CANX HIPK2 GPER MAPK15 SHARPIN AKT1 LOC728802 CDC34 MTRF1 ANKIB1 SEC14L4 FRZB RPGR RRM2B C20orf94 RP1L1 CATSPER2 STARD9 GPRIN3 WDR18 HSD17B12 CDNF E2F6 TMEM126B DPY19L4 CTPS2 PGBD2 RSRC2 CYP2B6 NES FAM115A FZD6 CUX1 ERCC6L PON2 MUC5AC TBCK ANKRD27 KRTAP9-7 UCHL5 ZNF28 PIP5KL1 AIM2 PNO1 HPS5 ANK3 SPDYE2 PTP4A1 C20orf108 CLRN3 QRSL1 UBR2 SREK1 FCRLA OR11A1 ZNF714 PIGW FAM48A MT1H PSMA8 C9orf80 OAZ1 HIST2H2BF SGCE YIPF5 WNT4 PTPLAD2 CSN2 TCF7 MIOS RLIM SAMD9 DTWD1 STRN4 HDGF SV2C LCN2 ZNF490 ARID4B GKAP1 OR1S1 RNF19A SPARC LHFPL3 KCTD12 CBX5 ZNF326 UBE2V2 ASB7 COL5A1 FASTKD1 FBXL18 WDR52 IFT57 TMEM2 ZNF592 RABL2A TNFSF9 CFP ZNF37A DOCK3 FAM208A STX16 PAQR9 ALDH3B1 ZNF266 DLAT FEZ2 OCR1 LMNA SLC9B1 TBCD IGIP ZNF250 TMEM48 APOA1 TATDN1 WNT5B TPSD1 TRIM59 ZNF12 PPP1R18 FTCD TTLL8 UHRF1 DNAJC24 WNK1 AHNAK2 WNT9B RFX7 TSHB HAS1 MEX3C TRPS1 C11orf46 SUPT7L PDIA4 ATP5E ATCAY SERBP1 TESC GTF2H3 ATG16L1 ERN1 JUN CSF1R PCNXL2 ATAD5 PTH1R B3GALT1 C1orf116 ADAMTSL3 C18orf10 VTA1 PNN CA6 ESPN CEP57 RBM43 MTMR3 TRNP1 KIAA1267 HIST1H3F DDX3X CSAG3 GATM HNRNPL RUNDC3A CDKN3 RSPH9 TMEM168 CASP12 DHX36 E2F3 FRG2B PHTF2 NUDCD1 HDC CCDC112 C2orf16 TFF3 SCGB3A2 NDFIP1 ATP6V1B1 GOLGA4 SULT1C4 PTPN13 OCA2 ZNF816 TMF1 TAS2R20 CELF4 CDKAL1 ST8SIA6 RPS6KC1 CDH5 STXBP3 SELENBP1 GOLT1B TMED4 MIB1 PCDHGB1 ZNF600 CES1 SMARCE1 TTC16 STK17B ZNF678 ICAM5 ADD2 ARHGAP27 TMEM106B METTL19 LRIG2 ZAK SKA2 USP1 SMEK2 C11orf54 IL7 ATMIN ZNF420 TTC7B SCN9A BTG3 RBL1 APP TOB1 ATP2B1 MS4A14 CENPF LOC100288602 SLC16A10 C12orf73 MDM4 NBPF15 HHATL KRT23 PNKD FAM21C CDX1 UBC PRR3 DPCD ZNF788 HBA2 HAPLN1 SNCB PUM1 KLHL24 CCDC88A PDE12 SGPP1 CNOT6L PMS2 HELQ BIRC3 AMZ1 ZNHIT6 WDR63 SPEF2 DCAF8 FAM161B HIST1H2BF WDSUB1 ZNF638 TMEM239 C6orf162 KCNK16 INSL3 DLG1 MT1B PINK1 KRTAP4-12 CNTNAP1 CYP26A1 ZNF684 ZNF492 SLC4A1 RTKN2 PDHA1 PRSS33 COX7A2L ANKRD36B RNF38 ARRB2 AHDC1 KLHL2 U2SURP MPL TMEM151A C9orf78 HERPUD2 CSPG4 IGF1 ZNF572 G3BP1 HSD17B2 NT5DC1 POLK ADCY7 PAK1 RBM15 AHSA2 TAS2R43 PLA2G2C PPP1R14A DCAF5 CYCS GJD3 TECTA ATP9B HMGCS2 PRKRA MITF BTN2A1 PAIP1 TAF9B C18orf12 SH3BP4 SLC33A1 UBB PON3 DCLK3 ANKRD13B USP47 TRIM33 G2E3 TMCC3 SLC38A5 NWD1 CBWD7 CXorf26 PPP1R13B SYCP3 PAPOLA C1orf27 TMEM235 SLC16A14 C17orf50 SLC4A8 RAB3IP ZNF852 CHRM5 FGFR1 ITLN2 COX6C SPECC1L CLCNKA NSMCE2 ZNF208 TTC37 HIST1H4E TMEM39A ZNF181 PIKFYVE LRRN2 KIAA1009 ANP32E YEATS4 SYNGAP1 BET1L NOC4L FRY C10orf12 ZNF718 PCYT2 UTF1 ATG2B RHOT1 ELOVL5 SHC1 ZNF673 BLOC1S2 CCDC64 HERC2 TMEM14E HOOK3 GINS1 CAND1 CLCC1 ENTPD3 MUDENG ASPRV1 ACSL3 KIAA1919 UTRN C20orf4 CDC14A CNN1 KRT1 LYZL1 INTS6 UCMA ZMYND15 A1CF GPR37L1 ZFP14 CECR2 NT5M TAS2R30 ZCRB1 DSCC1 KPNA3 EPHX3 DLX2 ZNF529 KATNB1 TSPAN13 FITM2 SPTSSA DUX4 BCLAF1 FOXJ1 GOLGA3 TRA2B SCUBE3 CDKL3 MTRR PPIL4 BBS7 KIAA1715 ZNF345 RTF1 KIAA2018 EXOSC2 CCDC57 YTHDF1 A2ML1 GTF3C3 HS3ST1 LMAN1L METTL2B CEP170 ZNF280C PF4 HECTD2 ZNF484 GOLGA6L10 C3 EMP2 SRSF6 SLCO2A1 FOXA2 PRDM16 SLC38A10 FRRS1 METTL20 RNF217 ATAD1 KRTAP19-1 UBR3 OTUD4 ZNF25 ATOH8 KBTBD3 YWHAQ FAM98B KIAA1704 SCGBL OR2T1 PPM1K HNRNPR SERPING1 KIR2DL4 STARD13 TMEM120B ZNF75A TNFRSF10A GPR114 SCP2 SON RWDD4 ALCAM ORAI3 FAM76B MZT1 QPRT TRAM1 ZNF605 UBXN4 ERLIN2 PPP1R9A OTUD6B ZNF41 TMEM117 ADAM19 PRPS2 GUCY2C TSPYL4 CELF1 TASP1 WDR19 EDNRA TMEM200A FYB GHRL MICU1 RAD18 RANBP6 FRA10AC1 FUCA2 PTMS CRYBA4 PIP4K2B PRRC2B BDH2 CRIPT DEPDC5 TRIP12 SMAD9 FGF8 SLAMF1 SENP7 LRRK2 MBD5 APPBP2 LIF PHLDA1 INO80D KDM5A SUV39H2 ERO1L FGF6 RASSF5 ZNF728 KIAA0317 TMEM87B IKZF3 LOC100652810 TMEM14C EZR ZNF549 ATF1 FGFR3 TMEM33 DPPA5 HINT3 ARIH1 BAGE BBS1 RGPD5 MFAP5 WDR20 C12orf4 CST3 WDR6 CHI3L1 CSDA VASH1 CNIH MOB4 SLC35A5 C9orf102 AKNA MIS18BP1 HPS4 KIF2A C9orf173 ARL6IP6 TTLL11 FAM71E2 HSPA8 UBXN7 DIDO1 ZNF320 CCDC18 C22orf13 CEL IMMT CNOT6 MARK3 PICALM STK40 AGGF1 PRRG1 EFHA2 ADAMTS2 PJA2 SLAIN1 C15orf59 NR2C2 NKTR NAALADL2 RBM44 C12orf59 FRYL ZNF432 OR8D1 UTP14C GPRC5C PAEP KCNJ13 VWA1 NOC3L C11orf68 FUBP1 NDNF OR10J3 PERP FBXO36 PBX1 UBL7 MTR SYTL1 FAM126B ALPP ZNF711 ASH1L PPP1R12A SPDYE5 VAMP4 RBM38 BCAP29 ZNF583 SLC35E2 ATP1A1 MTSS1L C5orf4 FRMPD3 IQSEC2 PLXNB3 EML5 UBQLN2 SORCS2 SETD8 ACVR2A NR1I3 PRIM2 YWHAZ SULT1A2 PGM2L1 KLHL9 KLRC1 OPA1 STRC RPL10 METTL21D IL1RL2 GPR119 MITD1 BCL2L15 WNT3A ZC3H6 PRPF39 DISC1 GABBR1 DZANK1 CLTCL1 LANCL2 NUAK1 ALG6 CDSN EFHA1 NPPA CATSPER4 ZFP41 OPN1MW VIT CARD14 ANKRD20A1 LCN1 RALGPS1 ARHGAP42 CCDC6 NCOA3 SPCS3 EFNA2 CLDN9 CHAF1A PHF21B PLA2G6 DEFB4A SEC63 MRPS28 VWA2 FTH1 MTRNR2L10 TOR1AIP1 SYTL4 VSTM4 SPRR2F ZNF107 MLL3 FOXL1 MOSPD1 PFKFB1 TMEM170A SVEP1 RAD1 COG4 ZNF354C C1orf198 HNRNPA0 OR2H1 ATP8B4 MEIS1 CCDC132 NGFRAP1 AMMECR1 MAGEC1 TTC30B SEC13 EGF HAGH VSIG1 NHLH2 TMEM179 BRCC3 USP17L2 TMEM82 FAM3B LOC100287633 KCND3 AQP8 ITGB1 CCL14 ZNF254 KCNG4 ZNF649 DNAJC16 SNX13 FAM100A NAIP SPATA12 OR1J2 XRCC4 SOX10 CHST10 O3FAR1 MAP1LC3B MEMO1 NAPB TRAPPC2 SOLH SVIP C1GALT1C1 SPAG9 OSBPL3 ZNF439 LOC100507096 SLC39A9 ANTXR1 EPB49 CRCP RHCE SENP8 TAC3 NKIRAS1 TCEANC KANK3 RAD54B PANK1 MED25 LOC100130345 FTSJD1 HS3ST6 ZNF442 ZNF157 EPS15 FDX1 FAM19A1 PLAC9 SH3BGRL2 ASPSCR1 PHOSPHO2 PEX26 ACBD7 PTGS2 CCDC90B UBE2D3 ADAMTSL4 SLC7A4 ARFGEF2 C4orf7 MAP1A SSC5D C17orf80 ZNF561 CTNNB1 ENTPD2 TRAPPC5 ANKRD17 STK3 REPS2 CHN2 ZNF781 VEZT CCNK CYP2C9 CNOT7 ESRP2 FSHR FCAR CST11 CD200 LCE2B MARCH3 NCKAP5L ORMDL1 WNK4 COL19A1 HNRPLL ITGA6 ZNF468 C1GALT1 THAP5 TMEM72 HNF4G IMPA2 LRCH4 MSR1 ZCCHC10 PDCD6IP PSIP1 SLC30A7 SLC17A4 SMCHD1 EIF2C3 PPP1R32 LAMC3 NET1 IPO5 UBE2Q2 CHDH PCDH15 ESR1 ARHGAP12 LGALS12 PGGT1B SURF4 C7orf41 PLEKHO2 LST1 ZNF790 NUDCD2 CNFN TPPP3 CHMP3 CILP2 SEL1L2 EDA2R DNA2 CEBPZ FAM107B RHOV DENND2A ANAPC1 KAT8 DMXL1 RBM3 APTX DMTF1 BMS1 CLRN2 GRM1 SCFD1 IBA57 RAPSN PDE4D  ROPN1L LRRC45 ZKSCAN1 TSPYL5 C4orf43 KCNMB3 PAPD4 PRKACB ZNF705A GNG2 TNFRSF10C HSD17B7 ZNF354A OR6Y1 PCDH11X JMJD5 TRANK1 RIMS1 OTOF CPB1 HIPK3 LOC645954 CDR2L GAL3ST1 CRAT FGFR4 PDZD7 ZWINT TRUB1 HNRNPCL1 PAK1IP1 TRIM14 MFAP3L ACAN FHL5 ZXDC TINAGL1 SMCR7 ALG11 ZNF780B DOCK4 ENOPH1 UBL4B SLC5A3 GAST PCDHB13 ZBED6 NAA15 SAR1A FRMD4A MCTP2 KRT31 ORC5 TNFRSF8 BIRC2 BCL11B CERS3 PTGIR ZC3H12C KIAA1841 PRM1 WDR38 C19orf51 TMEM53 HIST1H2AG PIGK S100A16 RNPC3 CCDC23 DPRX PFN4 KLF9 PYROXD1 C12orf32 OR1N2 ZFAND6 MAVS YES1 C6orf57 ABT1 HIST1H2BE NUF2 PWWP2A ARID2 PPP1R3F SMG1 FAM91A1 BTBD8 LFNG BROX ZDBF2 C17orf57 GNA13 POU2F2 TSPAN31 B4GALT4 FBXO25 NUDT16 OR10C1 MSTO1 CNKSR2 FUT8 TRMT11 BEND4 NEK10 FAM104A LRRC39 WWP1 SNURF C8orf73 ARGLU1 HIST2H2AC ZNF90 POLR2M OR10A4 EPHB1 POLD4 C8orf59 CASP8AP2 ANAPC10 C1orf9 FBXO30 NME4 HABP2 SERINC4 REEP1 UBE2T RPH3A C9orf156 CREM ATP2A3 POGLUT1 PTGER4 WEE1 SSFA2 BCL2A1 MTERFD1 SPAST RGS1 ACCN4 PRDM1 CCDC47 SMAP1 BSG PARP8 ZNF215 RERE FGFBP1 LINGO3 AJAP1 ALS2 ZNF608 TRIP11 COMMD8 CYTH4 NAP1L1 ECT2 FKBP8 GLCCI1 NPSA CCR4 KIAA1109 PTPRC B3GALT2 DCAF12L2 ZFX NUP35 ZNF727 C4orf45 IFT74 KLHDC8A BPIFB4 C9orf116 PNISR CARHSP1 ZNF548 CCDC102B ZNF577 NPAS2 FAM63A INPP5F IQGAP3 MYBL1 INPP5K CASQ1 CGREF1 SLC25A40 PDZK1IP1 ASB11 BUB3 C6orf211 VIL1 IFFO1 OR8U1 UBR1 CDC27 MDGA1 PTTG1IP ADARB1 ZNF664 GPC4 WDTC1 SIKE1 ZFP62 HEBP1 ZNF563 PARP15 UBASH3B C1orf114 GFPT1 ZCCHC6 SLC27A6 DTX2 KPNA4 H3F3A GLO1 MC4R CALML5 FAM126A TRPM7 INS SERPINB8 DYRK3 ALOXE3 NUCKS1 PPIL3 HNRNPD RBM34 CLEC4D GALNTL4 C12orf28 ANKUB1 KRTAP10-12 SMAD2 ODZ3 YWHAG ZNF555 IGF2BP2 FAM200A CENPE ZIC5 OR1D5 MCART6 TSSC4 CRABP2 COL11A2 CNTLN ACTR3B METTL14 TFAM PCDHA5 PUM2 ZNF365 ALG10B CDC14B SLC22A23 ARX C5orf63 NAT8B PPP3CB SRSF7 LSM14B PLCH1 FLCN MPZ NAMPT SSBP3 HECTD1 PCDHGB7 PRAMEF16 VKORC1L1 ABCA10 ZNF493 UROC1 NGEF ZNF582 RPS6 ZNF717 RSG1 MMD2 NEUROD2 C1QTNF9B OR4K14 KIDINS220 KIAA1644 THEM5 LIAS PGF CORT CTRC G3BP2 ISLR HNRNPA2B1 RNF216 DDX31 NHLRC2 ZNF430 AASS APOL5 ZNF793 RCHY1 RHOU PPP3R1 USP46 UGGT2 DMXL2 GNAZ PDCL DNAJC5G IGSF23 LILRB3 STIM2 SPI1 DCAF6 NAE1 FAM176B POTEF NUPL2 SPTY2D1 CD99L2 NKAIN4 PPP2R5B PTPRF IKBKB ZNF808 FRG1 MYEOV POLL PIP4K2A ZNF268 MED7 ZNF706 MYLK TFAP4 CYP4F2 PRIMA1 PGAP1 TMEM136 KRTAP2-1 OR2L2 ACSBG1 CTLA4 SNX9 TTC39A POLR2F ST6GALNAC4 GLMN KRT6C MCM7 C16orf47 DCLRE1A HIST1H4D SAMD11 TSC22D2 UBQLN3 TYW5 ASPG TLR7 SMAD1 ARHGEF15 CLCN1 RIOK2 PALM3 FAM18B2-CDRT4 ABHD13 CLEC7A HSPA14 CCPG1 TRDMT1 PDGFA ATP1A4 IL17F KRTAP12-4 C2orf43 KIAA1370 SLC38A3 BAZ2B PIGQ BRD7 POU6F2 GCOM1 KIAA0146 KRTAP9-1 PRSS55 PSMG1 CEP55 EYA3 KRT20 RPL34 AKAP12 AMBN KIRREL2 SLAIN2 GNE OR52N5 LRRC37B ZNF501 SLC22A31 ATF2 CTSE C10orf137 ARL6IP5 TNKS1BP1 CCNT1 IARS ARFIP1 PLSCR1 HELB PHACTR2 CD163L1 PLEKHG4B ZNF880 MAPK8 TSPO2 SYNCRIP WNT11 HDAC8 CBX7 STARD4 ZNF589 GPR35 MED13 NRGN DNAAF2 FAM179B LOC653550 TRA2A ESCO1 F2R MTAP ADORA3 COL6A5 TTI1 TRNT1 C10orf108 NSUN6 ACTL7A ANKRD44 DNAJC27 KIF27 CPNE6 EBF2 LACC1 CAPS2 CYP3A43 TMEM38B CCDC91 DNAJC21 PDE7A MRPS31 XPNPEP3 MTDH SPTA1 HSP90AB1 ZNF483 GEMIN2 PMAIP1 GOLGA6L6 MAPK13 RSBN1 GPR113 TBX18 C2 ERO1LB RALGPS2 SYT8 FER GSPT1 PIGY PMPCB NID2 SUZ12 EXTL2 LOC646508 PRSS47 ALS2CR8 PI4K2B LACRT MACC1 CXorf21 MFAP2 C10orf118 FAM72A OR1C1 ZNF415 SOCS1 TCEA1 MPP5 ZNF853 ZNF729 SERPINA9 CAMK4 TNFRSF14 C6orf174 CLPX HDGFRP2 ROBO3 ARSD CPLX2 FAM76A FGF5 RB1 ZFP2 CNIH4 C2orf56 GGCT TST SLC35D1 ERLIN1 TTC22 RNF24 CELF2 CD70 HIST1H2BK PF4V1 SHOC2 SRC FBXW7 MINA ERVH-3 ZNF606 TFAP2A SSR1 FAM48B1 NFS1 FAM163B OSBPL9 SLC25A36 DDX10 ZNF507 TMED10 CCDC87 LRRC8B CSNK1G1 ZNF26 ZNF169 GLIPR1L2 PHF8 PABPN1L ADIPOR1 GRINA RDH14 CLIP4 DNAJB2 PTPLB LUC7L3 RABEPK DPEP1 C14orf118 HAT1 HTR1D RAD51AP1 H2AFV MLL5 RC3H2 CXorf48 HKR1 TMEM87A BBS5 PHTF1 TMEM108 CMTM5 PKIA PQLC1 DDX59 GJC2 TNNI1 EIF2C2 SBDS CC2D2B GIPC3 MAN1B1 SP3 GPR17 UBE2Z ZNF772 RNASE1 ISOC1 TBC1D7 GRM7 VPS54 SLC39A8 MKI67 GLS DCAF10 IL4R KANK2 TMEM123 GLI2 TP53 C18orf32 CCDC69 ICA1 RAPGEF2 NAV3 MAP1LC3A SPAG1 ATP6V1G2 MNT C9orf82 KIAA1024 PPP6C PLGLB1 SMG7 ENTPD1 SPAG8 SH3BGRL3 ANKRD13A PURB ZCCHC11 C16orf87 ERVMER34-1 SYPL1 NLRP1 FAM71F2 LAMB2 HNF1B ZNF782 UMOD PPA2 MAP3K7 UPRT C10orf93 EID2 AKR1C1 ZNF615 BAG4 PPIP5K2 GTF2I KCNN2 ZNF32 DYNC2LI1 DGKE FAM53B ZNF746 CCDC54 ATXN1 TMEM59L KDM1B CPOX TIRAP KIRREL3 MADCAM1 FAM78B UBE2D4 TPM2 ELAVL1 NCOA2 S100PBP FAM170A BNIP3L ATP10D CFLAR C12orf56 GUK1 AGPAT5 PREPL RHPN2 C4orf39 SERF2 MRPS25 RABGAP1L KRT25 DLC1 PPP1R1A RAP2C RG9MTD2 AZI2 AHSG RBM39 RAB1A KRTAP15-1 ANKRD33 ELTD1 SHISA7 ZNF620 NFXL1 KMO VSIG2 FAM100B WHAMM RUFY3 C2orf3 MRPL42 KRTAP12-3 CD164 GFRA1 LMF2 SPHKAP TMEM211 ZKSCAN3 PLEKHG1 C1orf124 SP8 TPRKB COQ4 BRS3 PTPRU AFTPH ATP8B3 NHSL2 ADPGK ZNF418 LARP4 SIAE ZMPSTE24 DDX39B PFDN4 SNTB1 FAM181A KLHL11 SLC24A3 PSMB3 UBE3A TGFB1I1 MT1A ZBTB3 FAM57B TMEM192 DNM1L RIMS4 ORC4 RSPO3 DUXA EXOC7 TSPAN4 MPRIP TOLLIP DNAL1 FAM117B NLE1 SREBF1 IDE ANKRD32 PLVAP HMCN2 LTK OBFC2A GYG1 RAB18 ZNF91 SEMA6B MKRN1 KIAA1598 NXT2 WDR13 SERP2 TMEM184C APC FAM108B1 ZC3H15 ZNF233 WFDC1 ELK3 RRP15 GPR139 GALNT1 GPX1 PSG11 SBNO1 RSL1D1 P2RX7 ANKMY2 ADNP2 SNAPC3 RBM25 GNA12 ARCN1 PLEKHA5 TREML1 CDC42EP1 SEC24A KIAA0101 ZNF643 ZBTB25 TRMT1L MACROD2 IL18R1 MMP19 SPTBN4 AKAP11 FBXO28 MCMBP EPHA8 ACCN3 NAA11 EPHA10 MOCS2 ADAM11 ERGIC2 NAT1 CCDC7 HGFAC SPTSSB PEAR1 BACH2 FAM36A OR1S2 TFF1 FSD1L HAP1 EPHB2 LPHN2 MRPL30 PPARGC1B RNF123 COL27A1 ZFP42 TAS2R50 CCDC78 DEFB129 KDSR PHLDB2 MAP3K11 ZUFSP ESR2 MUM1 BHLHE41 ZNF280A EPM2AIP1 MID2 SRRM2 C7orf64 PCSK7 HNRNPA1 AMIGO2 TRIM36 BCAS3 TP53RK EVPL NOL10 OTOG HNRNPA1L2 EDEM3 RUFY2 TCOF1 SLC16A1 ADARB2 FAM45A GATC SF3B3 RANBP9 HSPB9 SLC17A5 NUDT21 C4orf46 INSIG1 SYNGR1 ACCSL PYCARD CDK3 ZNF235 ACSL4 FAM176A NKIRAS2 TRMT5 PPM1D RGPD6 ZNF850 CYP1B1 LOC100506127 C10orf32 AOC2 VGLL4 PDLIM5 XYLT1 BRWD1 ZFAT USP27X GOLPH3L STT3B SLC19A2 C9 ZBED5 B3GNT2 AP1AR LTA4H ZNF780A DUSP13 CD1C ZNF471 DCAF17 TMTC2 SLFN12L MCTP1 PDE3B L3MBTL1 FAM165B RPL39L ACER1 PCP2 CDYL RLF MLXIP NAT14 MTFR1 RRN3 HYAL3 SMNDC1 COBLL1 CLPB LTV1 SLC30A9 C7orf46 OTUD1 NDUFB1 OR1L6 ANKRA2 PVRL1 OCLM PANX1 RCBTB1 C12orf42 FGL2 WASL FAM178A CEP70 MYL4 SEPT14 TAF5L RGMB SOS1 ARHGAP11A PLAU TSTA3 NFE2 TOMM20 CTNND2 PTEN FRMD3 AFF1 ZNF189 AGFG1 GTF2A1 GUCY1A3 C2orf73 TC2N ODZ2 SENP1 MATR3 MRPS35 RBFOX3 CA13 RASSF3 MSC BTLA AASDH TFDP3 DCK LGR6 TIA1 SLC6A4 TXNDC15 SPTLC1 CALR3 FAM151B HLTF PKP1 C7orf58 SYNE2 C9orf85 GPR180 NDUFAF3 VCPIP1 NETO2 ODF3 LDHD SERPINA6 GALK1 ARL1 SOX13 SDCCAG8 KEL SSH2 EXOC5 FSCN2 PRAM1 GK RPL36A AK3 PTPDC1 AKD1 DUSP7 KDELC2 CEP135 TRIM22 GPR162 LYRM2 RC3H1 OR1E1 METTL8 TAF7 VEPH1 PRDX6 AKAP7 SLC25A26 CROCC AIDA ASCC3 GPX7 TGM2 STAM SLC22A18 RNF141 DNAJC22 ARHGEF7 OR7C2 TBK1 STX2 PKD2 FAM22A LIN54 CRLS1 C16orf59 CYP2U1 AADAC ZNF879 LRRC8C SAMD12 CCL3L3 KIAA1430 SEPT5 LOC390937 ZNF197 GABPB2 DACH1 CSGALNACT2 RNF208 ZACN PTPN11 KDM5C CALML4 APOL6 SLC2A13 MLL KIAA1958 SYT2 ATP8B2 LOC100133315 DICER1 ARMCX5 CNOT4 AMN1 ZNF141 HIF3A PSMC2 CAPZA2 KCNA2 ZNF749 PLA2G2A FOLH1 SFR1 SEC16B SPRR2D THOC2 TAF1B GPR173 MFAP3 GAN PCMT1 VAMP1 HDAC5 SLC25A32 YWHAE WDR1 GON4L ATP1B1 SWAP70 C5orf15 PTH2 NUP62CL LILRB4 ADAT2 REEP3 MGST3 ZNF596 CDKN1B LMBRD1 HIST1H3B BTRC LSM14A SLC25A16 STEAP3 EIF2B3 OR51F1 YEATS2 TRIM3 CABP5 RBM4 CSF1 PQBP1 REST SLC8A3 GMCL1 FNIP1 C9orf71 MRPL47 MGAT5 PLA2G4E IGSF1 TEAD3 SLC2A3 C2orf27A WDR43 ATP13A3 MYO5B FEM1B FOPNL HAO1 KIF5B PNMA5 TWF1 MAEL UHRF2 SDR42E1 MUC7 NUP153 MYH9 CENPH C9orf9 C3orf23 LRRC17 S100A5 PCGF6 RNF180 CPSF3L RAP1GAP MYEF2 TLR6 ZNF836 GAFA2 ASF1B SPOPL PACRGL C9orf66 MAGI3 ZNF812 CACNG7 KCNH2 TSEN15 CAMK2D MARCH8 CCDC122 TMEM161B TUBA3C ADCY5 SRPK3 FKBP14 TNNI3 HOXB3 SGCB NPAT ZNF547 MORN1 DUOX2 CDKL4 HAUS6 TMEM217 KIF16B CASP8 PRKD3 CFH RAB11FIP2 TSC2 RCOR3 C17orf110 NRIP1 BNIP2 C10orf81 GJA8 ACP2 CASC3 ACPT FLNC ARID5B AIM1L MAU2 MOG ZRSR2 KRAS SPTAN1 ERAP1 ATP7A CCNG2 ITSN2 RCAN2 LRRC69 PPARG BBS10 GYPE SPAM1 SIGLEC14 TANC1 HIST1H2AL CYP4F8 EVI5 ZWILCH SLC1A5 VPS41 SYCP1 IFT80 C15orf62 ZNF639 C19orf45 CACNG4 GFI1B ZNF407 C11orf58 PPIG SUN1 C12orf10 MRE11A CASD1 ZNF527 TTC35 SMC4 TTBK2 RASGEF1C PLXNC1 CTXN1 POMZP3 ZNF441 NUPL1 NRAS RPL22L1 ZNF138 MGAT5B FAM208B DUSP26 HDAC9 NYX RLN1 DNAJC3 C1orf21 IL36B TOM1L2 METTL15 TMEM165 CLDN24 RAB21 MUC8 CRY1 C12orf29 C10orf76 IL17RE CUZD1 PBRM1 CPB2 UHMK1 ELOVL7 FGF19 CHST13 GOLGA1 PIM1 USP9X EGFL7 VAPA TIGD1 STAP1 SLC34A1 FAM169A NEK7 PARP11 LEFTY1 CCL27 ACTR6 TMEM171 MYBPH CHCHD7 TCEA2 KRTAP5-4 PPP1CC YY1 ZNF92 CDCA7L CCDC138 AASDHPPT UBL4A LOC100130916 LRRC66 FAM110A SLC19A3 CDC37L1 ZNF585B C2orf82 SNRPE C8orf83 CARD6 STK38L LEPROT STEAP4 TNFRSF4 MTHFD2 TTC25 SLC27A1 CBLB OR6C74 AP2M1 STAC2 SLC9A7 POU5F2 SLC22A11 HMX2 NPRL3 P2RY1 STARD3 LIMA1 ZNF236 TAS2R19 GABRB1 MORF4L2 RPL7 FBLN1 MUC12 MRRF MYOF MTFMT TMTC3 LUC7L PRKAB2 ZNF76 QPCTL SUB1 MRPS33 ZIC1 DEFB124 AKTIP KLRK1 SLC16A9 ARL10 KIF20B RAMP1 ANKRD46 SNX4 CCR2 PDE8A RSPH1 LMBRD2 SYT15 SYNRG SMOX COPS2 KRT76 SDC4 AVIL FHL2 C11orf85 SUGT1 LOC375190 HIGD1A GOLGA8A SNRPD1 LPCAT2 DES CCDC158 BZW1 ASPA ITGAV ZNF221 CLDND1 MAGIX GDAP1 GH2 BTBD2 ACRBP MMACHC RBBP4 REV3L DUOXA1 PDPR PHKA1 ATG5 AVPI1 JHDM1D RPL22 RAD52 MORN3 CD207 B4GALT2 HMP19 KIAA1147 SGTB FEM1A SLC38A8 CHUK C9orf30-TMEFF1 RAB6B TTBK1 SCGN C11orf95 SRGAP2 MS4A5 DCST2 ZCCHC7 GPN3 MPP1 PCDHB4 CD2AP MSGN1 ZFP30 CCDC86 SMN2 RPS26 CTBP1 ZNF234 SPATA22 MYLK4 THOC1 KCNC3 STXBP5 CWC27 USP25 ZNF124 C3orf24 FAM18B2 KIF5A PCYOX1 ASCC2 KLHL35 SLC44A1 FBXO17 OPTC SPRED1 BABAM1 HBD PCK1 RAD51C ZC3HAV1L GPR45 ADAM10 ALPPL2 SPEN REV1 PRPS1 GALC NAP1L4 GNG4 TBKBP1 IQCF5 TTC32 ZNF217 LOC400927 LRRC34 AGPAT4 ZNF167 NOX4 ZBTB26 VWF TPM1 PYHIN1 PDS5B ZNF644 ZNF546 ZNF101 WDFY4 SCAI OPRL1 IQCG C14orf162 IL15 TECPR2 PLEKHA1 WDR78 LIG4 PFKFB3 ECSIT OSTM1 HAPLN2 OTP RNF168 LCTL NAPEPLD GALNT7 CCNYL1 KIAA2026 OBSCN SPECC1 HRASLS5 TRIM61 FAM155A DPY19L2 LRRC3C RRBP1 KLC3 IL1RAP MAT2B MTMR10 PRDX5 CLDN17 MED6 ERLEC1 CD46 PRMT6 ZKSCAN4 RBM12B C17orf48 OR5H6 ADRB2 PMFBP1 GABPB1 PRAP1 PRSS3 ERI2 C20orf46 HPGD TRERF1 FKBP1B FAM86C1 DRGX SLC24A2 MTBP ARHGAP19 ZNF252 VAV2 S100A6 PTCH1 TADA2A LOC388210 NEDD1 EPC1 DDX46 VPS13A SPRR1A TES PRPF38B SNX20 OSBPL6 AVPR2 KCNK12 GOPC GRB14 PNPLA8 RP2 NTRK3 C16orf52 NANP ZNF621 SEMA4B SNX14 FANCF CCNC HYDIN TADA1 SLC10A2 MCEE TNFRSF13C UBAP1 SLC35E2B ZFP82 ANKRD9 PBK MB21D1 PRRC1 DIS3 AP3B2 HSDL1 CDRT15L2 LCNL1 TMEM184B HNRNPH3 DIS3L2 ZNF292 SPIN1 EFCAB3 ARNTL PRB3 FUZ ZNF813 PUS10 KIAA1539 C4orf40 TMEM41B C16orf86 SPATA7 S100A11 CEP95 EFHC2 CLTC CLASP2 SRP9 PIN4 ZBTB8A RSBN1L PDE1A GPR88 RPRD2 SIGLEC6 DCTN6 GALNT4 TMEM194B PSME4 ZMAT3 CCDC114 MED23 ATF7IP FAM131A EEF1A1 ANP32D FAM151A CHAC1 MAST1 HMGA2 PRLHR RPF2 EIF4G1 UBE4A ZNF558 FAM169B MX2 C11orf30 RAB39B ICK SEL1L EIF2C1 DYRK1B NDUFC2 EFCAB4B C7orf51 ACSM5 PARP1 SIAH1 FAM60A BAG6 EIF4B BTF3L4 GZF1 C1orf95 OR9A2 SSPO RIN1 ITGA4 OR2G6 SEC23A PHLDB1 CLOCK TMEM86B CEP57L1 CPNE8 C14orf135 ROCK1 MCTS1 NCAPH TAGLN SHD ZC3H13 ZNF45 GPX6 ACYP1 OR9Q2 ZNF610 GAPVD1 C10orf88 TMBIM4 KLF1 ZNF140 FAM183A LOC100129216 BRD9 GLUD1 RLN2 OGT CAMKMT LSM11 APIP C7orf10 MSL2 MMP28 MARCH2 SREK1IP1 FAM35A HMGN5 ZNF701 HIST1H1B SMCR7L NBEAL2 PHF17 TYROBP PRKRIR TCP11 HSPA13 TMEM158 FASTKD3 RARA RMI1 FAM108C1 SS18 CARM1 MTRNR2L2 COTL1 NPHP4 IKZF2 DOCK9 PML TAF1A CBFB SESN3 HIST1H3H PPP1R12B ARL9 SOCS7 PDE2A FAM101A SACS AGBL3 ASPM TLE1 KRIT1 MGLL PIAS2 IL2RA PLDN PNRC2 CD24 NSF SLC22A17 KIF2B ANKRD20A2 NFASC CLCA4 ZNF597 LONRF3 SMURF1 HNRPDL PRIM1 TMSB15B CRYAA PLEK2 ACADSB HTR1F C14orf49 ZFP91 CEP78 SP100 UGDH LIME1 C2orf29 PLCB1 SRXN1 RPS20 LAMB1 CPEB2 TMEM56-RWDD3 IGF2 ATAD2 BPTF ADM2 NPEPPS CHRNA1 CD80 GPR77 JKAMP PHF6 HMGB4 GLYATL3 C10orf91 HTR1B MLLT6 PCGF5 RAP1B ZNF571 WIPF2 BRIP1 ZNF283 RASGRP1 CDH9 ZNF699 PPM1B PCF11 SLC7A5 FOXRED2 CYP20A1 NFIB AK4 CAPRIN1 ZNF23 FGF7 IL37 NPC1L1 UMODL1 TRIM13 CYGB SNAP23 CLECL1 GSTT2B GP9 ZC3H12D C7orf59 CYP4Z1 KCNAB2 MGA SLC35D3 MXI1 SRSF10 IL3RA TSEN2 MAG NELL1 MAP3K2 CEP97 ANKRD13C PCDH19 UBA6 SEPX1 JMY ZNF451 ADORA1 HES6 LEPROTL1 REEP5 POLE2 IL2 NOTO NIN PLCL1 ABI1 C2orf62 ZNF322 GRIN2D CASC5 IFIT5 GPR152 HOMER1 MCM9 C11orf87 ZNF286A ZNF302 CCDC101 OR1A1 PTGR1 ANKFY1 CNTRL ZNF69 OR7G2 CMPK1 SCN2A VHLL SSH1 C4orf21 RIBC2 ZNF449 GNAQ XPA DOCK10 CKAP2 FAIM2 CCNG1 PRB1 SPDEF ABCC10 PFDN6 TET2 EPG5 SRSF1 CNTNAP3 C5orf44 EEF1E1 DDN C12orf35 FNDC3A ESF1 ST8SIA3 MDK C20orf165 NCR1 ZNF14 PPIL2 DNAJC2 AP4S1 UBA52 MTRF1L CPNE3 C8orf38 PRKD1 TNFAIP8 ATP8A1 LRRC4B ANKRD50 CALCRL RECK TRIM54 RBM47 ZNF674 BCL2L1 BRSK2 C19orf77 RAB3IL1 EIF2A C17orf109 RAPGEF3 TMEM188 GBP6 MAPK3 SLC20A2 KIAA1549 ZDHHC23 CRBN SLC16A3 ZNF367 NCBP1 FGFR1OP2 DNAJC28 PRR7 KIAA1826 ELSPBP1 FOXF1 GTPBP10 CRYZ DNAJB9 PAFAH1B2 EDC4 WBP2 UBAP2L MPZL3 KCNK15 PRPF18 SMOC1 C10orf58 MOB1A GPR146 SPC25 TMEM234 WDHD1 EIF3C OR5A1 SCRN3 C19orf33 CRNN TMEM231 SLC45A4 HSH2D TRIM32 SCAMP1 PTS EID3 DUX4L4 MC5R NXF3 TMEM170B TAS2R40 KIF15 COPG ZNF569 GDF5 WFDC11 FGFR1OP OR2T8 MED28 GOLIM4 ALPI HSF1 ZNF273 DST DR1 NPSR1 SLC17A2 GLRX5 CAB39 HOXA9 MSH2 LNX2 ZEB2 CYLD MS4A18 POLR2H KRTAP23-1 WTAP WSCD2 ABCG5 KIAA1468 MGAT4A ZNF180 JMJD1C LOC730755 CHRM2 STK35 USP48 CAPN5 RRM1 OLFML2B NAA30 CTDSPL KLHDC5 ABHD11 CADM1 POLR3H KLHL29 RILP PAIP2B TSPAN9 WDR67 KCNRG THUMPD1 ARID4A PPAPDC2 KLHDC10 SYCP2 THUMPD2 LGI3 CALM3 TSPAN1 RGS9 ZNF100 SPATS1 SRSF2 NKAPL AQP5 RAB30 CEP41 SDHC MS4A7 OSBPL11 YLPM1 GNB5 CCL21 OR1F1 PRKAR1B RPTN ZFP112 ZC3H11A UBTD2 FYTTD1 IQCJ CCDC88C |
| Intersection | 27 | RPS17 NDUFS4 RPL39 MRPS18C NDUFB3 DBI EIF3E RPL31 COMMD6 SLIRP EEF1B2 RPL26 SHFM1 TOMM7 LSM3 SNRPG RPL17 RPS24 HSPE1 RPS27L COX7C ATP5I RPS27A HSP90AA1 RPL21 RPL23 RPS27 |
