# Supplementary material for: Differential Expression of mRNAs in Peripheral Blood Related to Prodrome and Progression of Alzheimer's Disease
Source: Biomed Res Int. 2020 Oct 31;2020:4505720. doi: 10.1155/2020/4505720 (PMC7648929; doi:10.1155/2020/4505720)
Supplement: Supplementary 2 — Supplementary Table 2: KEGG pathway analysis of DEGs in the MCI group. [file 4505720.f2.docx]

| Term | P-value | Adjusted P-value | Combined Score | Genes |
| --- | --- | --- | --- | --- |
| Ribosome | 1.21E-27 | 3.73E-25 | 1809.098 | RPL5;RPL41;RPL21;RPL31;RPL23;MRPS21;RPS27L;RPS3A;RPL9;MRPS18C;RPS25;MRPL3;RPS17;RPS27;RPS15A;RPL36AL;RPL35;RPL26;RPS27A;RPL39;RSL24D1;RPL17;RPS24 |
| Oxidative phosphorylation | 1.76E-13 | 2.71E-11 | 557.3748 | ATP6V1G1;COX7B;NDUFA4;NDUFB3;NDUFB2;NDUFA1;COX7A2;COX7C;UQCRH;UQCRHL;UQCRQ;NDUFS5;NDUFS4 |
| Parkinson disease | 9.02E-12 | 9.26E-10 | 417.3094 | COX7B;UQCRQ;NDUFA4;NDUFS5;NDUFS4;NDUFB3;NDUFB2;NDUFA1;COX7A2;COX7C;UQCRH;UQCRHL |
| Non-alcoholic fatty liver disease (NAFLD) | 1.60E-11 | 1.23E-09 | 388.7757 | COX7B;UQCRQ;NDUFA4;NDUFS5;NDUFS4;NDUFB3;NDUFB2;NDUFA1;COX7A2;COX7C;UQCRH;UQCRHL |
| Alzheimer disease | 8.06E-11 | 4.97E-09 | 316.6886 | COX7B;UQCRQ;NDUFA4;NDUFS5;NDUFS4;NDUFB3;NDUFB2;NDUFA1;COX7A2;COX7C;UQCRH;UQCRHL |
| Thermogenesis | 1.98E-10 | 1.02E-08 | 244.129 | COX7B;COX16;NDUFA4;NDUFB3;NDUFB2;NDUFA1;COX7A2;COX7C;UQCRH;UQCRHL;UQCRQ;NDUFS5;NDUFS4 |
| Huntington disease | 3.29E-10 | 1.45E-08 | 263.6227 | COX7B;UQCRQ;NDUFA4;NDUFS5;NDUFS4;NDUFB3;NDUFB2;NDUFA1;COX7A2;COX7C;UQCRH;UQCRHL |
| Cardiac muscle contraction | 3.06E-06 | 1.18E-04 | 189.6297 | COX7B;UQCRQ;COX7A2;COX7C;UQCRH;UQCRHL |
| Retrograde endocannabinoid signaling | 1.18E-04 | 0.004023 | 71.22927 | NDUFA4;NDUFS5;NDUFS4;NDUFB3;NDUFB2;NDUFA1 |
| Proteasome | 0.001609 | 0.049547 | 83.26658 | PSMA6;PSMC6;PSMA4 |
| Spliceosome | 0.005107 | 0.143006 | 30.58731 | SNRPD2;SF3B6;SNRPG;LSM3 |
| Mitophagy | 0.04429 | 1 | 18.62285 | TAX1BP1;TOMM7 |

Supplementary table 2
