# Supplementary material for: Differential Expression of mRNAs in Peripheral Blood Related to Prodrome and Progression of Alzheimer's Disease
Source: Biomed Res Int. 2020 Oct 31;2020:4505720. doi: 10.1155/2020/4505720 (PMC7648929; doi:10.1155/2020/4505720)
Supplement: Supplementary 3 — Supplementary Table 3: KEGG pathway analysis of DEGs in the AD group. [file 4505720.f3.docx]

supplementary table 3

| Term | P-value | Adjusted P-value | Combined Score | Genes |
| --- | --- | --- | --- | --- |
| Ribosome | 4.96E-20 | 1.53E-17 | 1387.597 | RPL21;RPL31;RPL23;RPL11;MRPS21;RPS27L;MRPS18C;RPS25;RPS17;RPS27;RPL36AL;RPL26;RPS27A;RPL39;RPL17;RPS24 |
| Oxidative phosphorylation | 6.92E-13 | 1.07E-10 | 691.2663 | NDUFA4;UQCRQ;NDUFS5;NDUFS4;COX17;NDUFB3;NDUFA1;COX7A2;COX7C;UQCRH;UQCRHL |
| Parkinson disease | 4.15E-11 | 4.26E-09 | 502.5294 | NDUFA4;UQCRQ;NDUFS5;NDUFS4;NDUFB3;NDUFA1;COX7A2;COX7C;UQCRH;UQCRHL |
| Non-alcoholic fatty liver disease (NAFLD) | 6.70E-11 | 5.16E-09 | 469.3334 | NDUFA4;UQCRQ;NDUFS5;NDUFS4;NDUFB3;NDUFA1;COX7A2;COX7C;UQCRH;UQCRHL |
| Alzheimer disease | 2.61E-10 | 1.61E-08 | 385.2039 | NDUFA4;UQCRQ;NDUFS5;NDUFS4;NDUFB3;NDUFA1;COX7A2;COX7C;UQCRH;UQCRHL |
| Thermogenesis | 2.80E-10 | 1.44E-08 | 312.6742 | NDUFA4;UQCRQ;NDUFS5;NDUFS4;COX17;NDUFB3;NDUFA1;COX7A2;COX7C;UQCRH;UQCRHL |
| Huntington disease | 8.53E-10 | 3.75E-08 | 322.9813 | NDUFA4;UQCRQ;NDUFS5;NDUFS4;NDUFB3;NDUFA1;COX7A2;COX7C;UQCRH;UQCRHL |
| Cardiac muscle contraction | 6.33E-06 | 2.44E-04 | 229.0394 | UQCRQ;COX7A2;COX7C;UQCRH;UQCRHL |
| Spliceosome | 8.67E-05 | 0.002967 | 104.1774 | SNRPD2;ZMAT2;SF3B6;SNRPG;LSM3 |
| Retrograde endocannabinoid signaling | 1.38E-04 | 0.004264 | 89.60288 | NDUFA4;NDUFS5;NDUFS4;NDUFB3;NDUFA1 |
| IL-17 signaling pathway | 0.038905 | 1 | 20.84173 | HSP90AA1;S100A8 |
