# Supplementary material for: Differential Expression of mRNAs in Peripheral Blood Related to Prodrome and Progression of Alzheimer's Disease
Source: Biomed Res Int. 2020 Oct 31;2020:4505720. doi: 10.1155/2020/4505720 (PMC7648929; doi:10.1155/2020/4505720)
Supplement: Supplementary 4 — Supplementary Table 4: KEGG pathway analysis of DEGs in the advanced AD group. [file 4505720.f4.docx]

Supplementary table 4

| Term | P-value | Adjusted P-value | Combined Score | Genes |
| --- | --- | --- | --- | --- |
| Herpes simplex virus 1 infection | 3.18E-20 | 9.80E-18 | 74.38853 | TBK1;ZNF729;ZNF606;ZNF727;ZNF605;ZNF726;AKT3;ZNF846;ZNF845;AKT1;ZNF721;ZNF600;ZNF841;ZNF169;ZNF17;ZNF284;ZNF283;ZFP1;ZNF160;ZNF705A;ZNF10;ZNF12;HCFC2;ZNF14;ZNF718;ZNF717;ZNF836;ZNF714;ZNF713;TP53;ZNF157;ZNF155;ZNF154;ZNF273;HKR1;ZNF23;PIK3R1;CFP;ZNF25;ZNF26;C3;ZNF709;ZNF823;ZNF701;ZNF700;ZNF268;ZNF267;ZNF141;ZNF383;ZNF382;ZNF140;EIF2AK2;BST2;ZNF37A;ZNF816;ZNF30;ZNF813;RBAK;ZNF257;ZNF254;ZNF253;ZNF250;ZNF492;ZNF490;ZNF45;IKBKB;ZNF41;ZNF248;JAK2;IKBKE;ZNF124;ZNF486;ZNF484;ZNF480;ZNF780B;ZNF780A;ZNF235;ZNF234;ZNF597;TLR3;ZNF233;ZNF596;ZNF595;ZNF230;SP100;ZNF471;ZFP14;ZNF107;ZNF226;ZNF468;ZNF347;ZNF589;ZNF225;ZNF585B;ZNF587;ZNF222;ZNF101;ZNF221;ZNF100;ZNF583;ZNF461;ZNF582;ZNF460;IFNB1;ZNF33A;FAS;ZNF699;ZNF577;ZNF354A;ZNF333;ZNF354C;ZNF354B;ZNF571;ZNF570;PPP1CB;PPP1CC;ZNF81;ZFP30;ZNF83;ZNF84;ZNF208;ZNF85;ZNF569;ZNF568;ZNF688;ZNF567;ZNF566;ZFP37;MAP3K7;ZNF443;ZNF684;ZNF563;ZNF442;ZNF562;ZNF320;IFNAR2;ZNF561;ZNF682;ZNF680;TSC2;ZNF98;ZNF99;ZNF90;ZNF91;ZNF93;ZNF439;ZNF559;SRSF2;SRSF3;ZNF558;ZNF799;ZNF436;SRSF6;ZNF555;ZNF676;SRSF7;ZNF675;ZNF432;ZNF674;ZNF431;ZNF551;ZNF793;ZNF430;ZNF550;ZNF670;ZNF791;ZNF790;IRAK1;ZNF429;ZNF549;ZNF669;ZNF548;ZNF547;ZNF546;ZNF667;TNFRSF14;ZNF302;ZNF543;ZNF300;ZNF420;ZNF540;ZNF782;EIF2B3;POU2F2;EIF2S1;POU2F3;MAVS;ZNF419;ZNF418;ZNF416;ZNF658;BCL2;ZNF415;CYCS;TAB2;BCL2L1;ZNF772;ZFP82;CASP8;ZNF529;ZNF649;CASP3;ZNF527;ZNF765;CHUK;IRAK4;PIK3CA;ZNF519;IFNG;TRAF3;ZNF517;ZNF879;ZNF878;LTA;BIRC2;BIRC3;ZNF195;SRC;SRSF1;NXF3;ZNF749;ZNF746;ZNF286B;ZNF624;ZNF623;ZNF621;ZNF620;NXF5;ZNF286A;ZNF189;ZNF184;ZNF181;ZNF180;PTPN11;PML;ZNF737;ZNF615;ZNF736;ZNF614;ZNF853;ZNF610;ZNF850 |
| p53 signaling pathway | 0.001249 | 0.192327 | 10.63516 | STEAP3;GORAB;PTEN;RCHY1;PPM1D;CASP8;CCND2;SESN3;ZMAT3;CHEK2;CASP3;SESN1;PERP;PMAIP1;SFN;IGFBP3;SIAH1;TSC2;IGF1;RRM2B;CCNG2;CDK2;CCNG1;BCL2;CDK1;MDM2;FAS;CYCS;ATM;MDM4;TP53;ATR;TP73;BCL2L1 |
| Ubiquitin mediated proteolysis | 0.001909 | 0.195979 | 8.775606 | UBE2D4;UBE3C;UBE2D3;UBE3A;CBLB;UBE2Z;CDC20;HERC4;CDC23;HERC2;UBE2Q2;CDC27;FBXO4;BTRC;SKP2;FBXW7;UBE2E1;UBE4A;UBE2E2;PIAS2;DDB1;CDC34;ANAPC4;BIRC6;ANAPC1;BIRC2;BIRC3;CUL5;UBA6;CUL3;XIAP;RCHY1;ANAPC10;UBE2J1;SOCS1;UBE2NL;UBR5;UBE2I;PPIL2;UBE2B;SMURF2;FANCL;SMURF1;SIAH1;HUWE1;WWP1;PML;KLHL9;UBE2W;UBE2N;UBA3;MDM2;UBA2;ERCC8;TRIP12;TRIM32;CUL4B |
| Cell cycle | 0.004231 | 0.325823 | 7.57213 | RB1;YWHAE;CDKN1C;CDKN1B;MCM7;CDC14A;CDC14B;CDC20;CDC23;CCND2;YWHAQ;CHEK2;CDC27;SKP2;YWHAG;SMC1A;YWHAZ;CCNA2;RBL1;DBF4;TFDP2;ANAPC4;TP53;ANAPC1;TTK;PKMYT1;ANAPC10;ORC5;ORC4;ORC6;RAD21;ORC3;ORC2;ABL1;E2F2;SFN;E2F3;BUB3;E2F5;SMAD2;SMAD4;MAD2L2;STAG1;WEE1;STAG2;CDK2;MDM2;CDK1;ATM;ATR;MAD2L1 |
| Prostate cancer | 0.009928 | 0.61159 | 6.407283 | RB1;CDKN1B;HSP90AB1;TCF7;LEF1;ARAF;PTEN;PDGFA;PIK3R1;FOXO1;INS;IKBKB;NRAS;PLAU;CREB3L1;AKT3;PDGFC;AKT1;E2F2;E2F3;NKX3-1;MAPK3;TCF7L2;HSP90AA1;CHUK;EGF;IGF1;MMP9;CREB1;ZEB1;PIK3CA;CDK2;MDM2;BCL2;CTNNB1;KRAS;SOS1;TP53;SOS2;FGFR1 |
| Proteoglycans in cancer | 0.011797 | 0.605587 | 5.58086 | ITGB1;ITGB5;ARAF;ELK1;ACTB;PPP1CB;PPP1CC;SHH;PLAU;CASP3;AKT3;AKT1;TIMP3;ITGAV;PRKACB;ARHGEF12;PPP1R12A;WNT5B;RPS6;GAB1;WNT9B;RRAS2;FRS2;ANK2;ANK3;ANK1;HSPG2;MMP9;VAV2;CTTN;PIK3CA;EZR;PPP1R12B;SOS1;TP53;SOS2;CAMK2D;SDC4;ROCK1;ROCK2;SRC;ITPR2;PIK3R1;HIF1A;NRAS;PAK1;WNT11;ERBB3;GPC3;FLNA;FLNC;EIF4B;WNT4;MAPK3;SMAD2;FZD3;WNT3A;CAV2;PTCH1;CAV1;FZD6;RDX;IGF2;PTPN11;IGF1;MAPK14;ESR1;MAPK13;TFAP4;RPS6KB1;MDM2;CTNNB1;FAS;KRAS;FGFR1 |
| TNF signaling pathway | 0.01313 | 0.577714 | 5.838458 | ATF2;CSF1;PIK3R1;PTGS2;CXCL2;CX3CL1;IKBKB;MAPK9;BAG4;MAPK8;RPS6KA5;CASP8;CREB3L1;CASP3;AKT3;AKT1;MAP3K8;DNM1L;MAP3K7;MAPK3;MAP2K3;MAP2K4;JUN;CHUK;IL15;IFNB1;LIF;DAB2IP;VEGFC;CFLAR;MAPK14;MMP9;MAPK13;CREB1;PIK3CA;TRAF3;LTA;TAB3;FAS;TAB2;PGAM5;BIRC2;IL18R1;BIRC3 |
| Small cell lung cancer | 0.013876 | 0.534244 | 5.88789 | RB1;ITGB1;CDKN1B;LAMC3;MAX;LAMA4;ITGA2B;LAMA3;PTEN;XIAP;PIK3R1;PTGS2;IKBKB;CASP3;AKT3;AKT1;E2F2;ITGAV;E2F3;POLK;SKP2;CHUK;NOS2;LAMB2;LAMB1;PIK3CA;TRAF3;COL4A4;CDK2;COL4A6;CKS2;BCL2;CYCS;ITGA6;TP53;BIRC2;BIRC3;BCL2L1 |
| Mitophagy | 0.01482 | 0.507157 | 6.111901 | USP15;SRC;HIF1A;MAPK9;NRAS;MAPK8;TBK1;RHOT1;UBB;MFN1;MFN2;TBC1D17;ATG5;BNIP3L;FIS1;JUN;CSNK2A1;ATG9A;TFE3;TFEB;RRAS2;MITF;PINK1;TOMM7;PGAM5;KRAS;TP53;BCL2L1 |
| Progesterone-mediated oocyte maturation | 0.02428 | 0.747826 | 4.934161 | HSP90AB1;PDE3B;ARAF;ADCY4;GNAI3;ADCY2;PIK3R1;PKMYT1;ADCY7;ANAPC10;ADCY5;INS;RPS6KA3;MAPK9;MAPK8;CDC23;SPDYE2;AKT3;CDC27;AKT1;PRKACB;MAPK3;HSP90AA1;IGF1;SPDYE5;MAPK14;MAPK13;CCNA2;MOS;MAD2L2;PIK3CA;CDK2;CDK1;ANAPC4;KRAS;CPEB2;ANAPC1;CPEB4;MAD2L1 |
| mRNA surveillance pathway | 0.027675 | 0.774892 | 4.780618 | HBS1L;SMG1;RNMT;CSTF3;CSTF2T;CASC3;MSI2;NXT2;SMG7;SMG5;GSPT1;PABPN1L;PPP1CB;PNN;PPP1CC;NXF3;PCF11;PAPOLG;PAPOLA;PABPC4L;NXF5;UPF2;NCBP1;PABPC4;PPP2R5B;SSU72;UPF3B;UPF3A;PPP2R5C;MAGOHB;WDR33;NUDT21;DDX39B;WDR82;PPP2R2D;PABPC1L |
| MAPK signaling pathway | 0.028946 | 0.742939 | 4.166465 | ATF2;CSF1;ARAF;IL1RAP;FGF1;ELK1;ELK4;FGF5;RPS6KA3;FGF6;FGF7;RPS6KA5;FGF8;AKT3;STMN1;AKT1;MAP3K8;PRKACB;MAP3K7;MAP2K3;MAP2K4;MEF2C;IL1R1;PGF;CACNB1;CACNB4;TP53;CSF1R;MAX;PDGFA;CACNA1F;RASGRP1;RASGRP3;STK3;RAP1B;PPP3R1;IRAK1;PDGFC;FGF23;HSPA8;JUN;JUND;IGF2;IGF1;EFNA1;EFNA2;FGF19;NF1;TAB2;FGFR4;FGFR3;MAP3K11;FGFR1;ARRB1;ARRB2;ECSIT;DUSP16;IKBKB;PPP3CB;MECOM;CASP3;DUSP4;CHUK;PLA2G4E;RRAS2;IRAK4;DUSP8;TGFBR1;DUSP7;PPM1A;PPM1B;RASA1;RASA2;RAPGEF2;SOS1;SOS2;INS;CACNG6;CACNG7;MAPK9;NRAS;PAK1;MAPK8;ERBB3;GNA12;FLNA;FLNC;CACNG2;MAP4K3;CACNG4;MAPK3;MAP3K2;NTRK1;EGF;VEGFC;MAPK14;MAPK13;TAOK2;FAS;KRAS;LAMTOR3;PTPN5;HSPA1A |
| cGMP-PKG signaling pathway | 0.029488 | 0.69863 | 4.362084 | ATF2;CALML5;PDE3B;ATP2A3;ATP2A2;CALML4;SLC8A1;MYLK;SLC8A2;MYLK4;PPP1CB;PPP1CC;EDNRA;PPP3CB;CREB3L1;ADORA3;AKT3;NPPA;ADORA1;AKT1;GTF2I;MEF2C;MEF2B;PPP1R12A;ATP1B2;ATP1B1;CREB1;ADRB3;KCNMA1;PDE5A;PLCB1;MYL9;CNGB1;ROCK1;ROCK2;ATP1A4;ADCY4;GNAI3;ITPR2;ADCY2;ADRB2;ATP1A1;CACNA1F;ADCY7;KNG1;ADCY5;INS;GNA13;PPP3R1;GNA11;GNA12;KCNMB3;MAPK3;PDE2A;ATP2B4;NFATC2;ATP2B3;ATP2B1;SLC8A3;GNAQ;CALM3 |
| Adrenergic signaling in cardiomyocytes | 0.030044 | 0.660978 | 4.397302 | ATF2;CALML5;ATP2A2;CALML4;SLC8A1;PPP1CB;PPP1CC;RPS6KA5;CREB3L1;AKT3;AKT1;SCN5A;PRKACB;TPM3;TPM2;TPM1;PPP2R5B;ATP1B2;PPP2R5C;ATP1B1;MYL4;CACNB1;CACNB4;CREB1;PLCB1;RAPGEF3;KCNE1;CAMK2D;CREM;ATP1A4;ADCY4;GNAI3;ADCY2;ADRB2;ATP1A1;CACNA1F;ADCY7;ADCY5;CACNG6;CACNG7;TNNI3;CACNG2;CACNG4;MAPK3;ATP2B4;ATP2B3;ATP2B1;MAPK14;MAPK13;PPP1R1A;GNAQ;PPP2R2D;BCL2;CALM3 |
| Pancreatic secretion | 0.033387 | 0.685553 | 4.440641 | PNLIPRP2;CELA3B;CPB2;CPB1;SCT;ATP2A3;ATP1A4;ADCY4;ITPR2;ATP2A2;ADCY2;PLA2G5;ATP1A1;SLC4A4;ADCY7;ADCY5;RAP1B;PRSS3;CLCA4;CPA3;SLC12A2;CPA2;CELA2B;PLA2G12B;PLA2G2C;TRPC1;PLA2G2A;ATP2B4;ATP2B3;ATP1B2;CEL;ATP2B1;ATP1B1;RAB11A;GNAQ;KCNMA1;PLCB1;SLC26A3 |
| IL-17 signaling pathway | 0.03858 | 0.742673 | 4.244581 | HSP90AB1;SRSF1;PTGS2;CXCL2;ELAVL1;IKBKB;MAPK9;MAPK8;CASP8;TBK1;CASP3;MAPK6;MAP3K7;IKBKE;MAPK3;USP25;HSP90AA1;JUN;JUND;CHUK;IL17RE;MAPK14;MMP9;MAPK15;MUC5AC;MAPK13;IFNG;TRAF3;LCN2;TAB3;IL17F;TAB2;DEFB4A;IL17C;S100A7;IL17A |
| Hepatitis B | 0.042655 | 0.772813 | 3.846558 | RB1;ATF2;DDX3X;ARAF;ELK1;IKBKB;TBK1;CASP8;CASP12;YWHAQ;CREB3L1;CASP3;AKT3;AKT1;JAK2;MAP3K7;IKBKE;MAP2K3;MAP2K4;CHUK;IRAK4;HSPG2;MMP9;YWHAZ;TIRAP;TGFBR1;CCNA2;DDB1;CREB1;PIK3CA;TRAF3;SOS1;TP53;SOS2;TLR3;SRC;PIK3R1;MAPK9;NRAS;MAPK8;IRAK1;E2F2;E2F3;MAPK3;SMAD2;EGR2;SMAD4;JUN;IFNB1;NFATC2;MAPK14;MAPK13;MAVS;CDK2;BCL2;CYCS;FAS;TAB2;KRAS |
| N-Glycan biosynthesis | 0.042759 | 0.731659 | 4.459864 | B4GALT2;ST6GAL1;B4GALT1;ALG6;ALG2;ALG13;ALG11;ALG10;DPM2;DPM3;FUT8;MAN1A2;MGAT5B;MGAT5;MGAT4A;MAN1C1;MGAT1;MAN1B1;MGAT2;STT3B;ALG10B |
