# Supplementary figures and images for: Differential Expression of mRNAs in Peripheral Blood Related to Prodrome and Progression of Alzheimer's Disease
Source: Biomed Res Int. 2020 Oct 31;2020:4505720. doi: 10.1155/2020/4505720 (PMC7648929; doi:10.1155/2020/4505720)

A

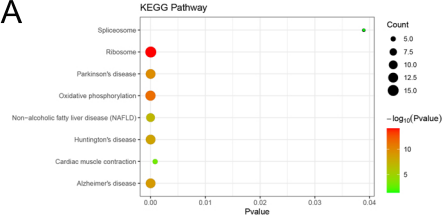

B

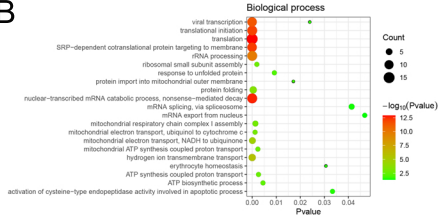

C

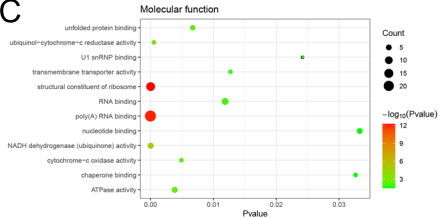

D

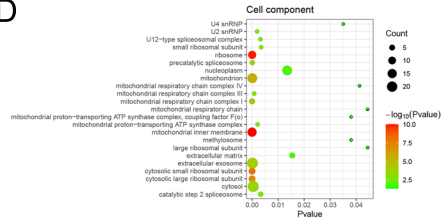

Supplement: Supplementary 5 — Supplementary Figure 1: the enrichment analysis of the intersecting genes between MCI and AD. [file 4505720.f5.pdf]
